# Validity and contributions to pain from the central aspects of pain questionnaire in rheumatoid arthritis

**DOI:** 10.1097/PR9.0000000000001295

**Published:** 2025-06-20

**Authors:** Stephanie Louise Smith, Vasileios Georgopoulos, Onosi Sylvia Ifesemen, Richard James, Eamonn Ferguson, Richard J. Wakefield, Deborah Wilson, Philip Buckley, Dorothy Platts, Susan Ledbury, Ernest Choy, Tim Pickles, Zoe Rutter-Locher, Bruce Kirkham, David Andrew Walsh, Daniel F. McWilliams

**Affiliations:** a Pain Centre Versus Arthritis, University of Nottingham, Nottingham, United Kingdom; b Advanced Pain Discovery Platform, University of Nottingham, Nottingham, United Kingdom; c NIHR Biomedical Research Centre, University of Nottingham, Nottingham, United Kingdom; d Academic Rheumatology, Academic Unit of Injury, Recovery and Inflammation Sciences, School of Medicine, University of Nottingham, Nottingham, United Kingdom; e School of Psychology, University of Nottingham, Nottingham, United Kingdom; f NIHR Blood and Transplant Research Unit in Donor Health and Behaviour, University of Cambridge, Cambridge, United Kingdom; g Leeds Institute of Rheumatic and Musculoskeletal Medicine, University of Leeds, Leeds, United Kingdom; h NIHR Leeds Biomedical Research Centre, University of Leeds, Leeds, United Kingdom; i Leeds Teaching Hospitals Trust Hospitals Trust, Leeds, United Kingdom; j Rheumatology, Sherwood Forest Hospitals NHS Foundation Trust, Sutton-In-Ashfield, Nottinghamshire, United Kingdom; k Independent Consultant, Nottingham, United Kingdom; l School of Medicine, Cardiff University, Cardiff, United Kingdom; m Centre for Trials Research, Cardiff University, Cardiff, United Kingdom; n Rheumatology Department, Guy's and St Thomas' NHS Foundation Trust, London, United Kingdom; o School of Immunology and Microbial Sciences, Faculty of Life Sciences and Medicine, Kings College London, London, United Kingdom

**Keywords:** Pain, Psychometrics, Arthritis, Rheumatoid, Surveys and questionnaires

## Abstract

**Introduction:**

The central nervous system (CNS) contributes to pain perception across musculoskeletal conditions. The central aspects of pain (CAP) questionnaire captures a single score associated with quantitative sensory testing (QST) evidence of CNS dysfunction validated in knee osteoarthritis.

**Objectives:**

Given the different pathophysiology of rheumatoid arthritis (RA), an inflammatory polyarthritis, this cross-sectional study assessed CAP's psychometric properties and its association with pain in RA.

**Methods:**

Adults with RA were recruited from Nottinghamshire, London, and Cardiff. Participants completed CAP and reported pain using a numerical rating scale. A subgroup underwent additional assessments, including quantitative sensory testing (QST; Pressure Pain detection Threshold, Temporal Summation, Conditioned Pain Modulation), Disease Activity Score-28, C-reactive protein, questionnaires addressing pain and related characteristics, and Central Sensitization Inventory short form (CSI-9). Cronbach alpha, confirmatory factor (CFA), and Rasch measurement theory assessed CAP's reliability and validity. Multivariable linear regression modelled contributions to pain by inflammation indices and CAP or CSI-9.

**Results:**

The 380 participants (73% female, median 63 years) reported average pain over the past 4 weeks of 6/10 and a CAP score of 9/16. Central aspects of pain demonstrated acceptable reliability (ICC_(2,1)_ = 0.71), CFA fit (comparative fit index = 0.99, Tucker–Lewis index = 0.99, root mean square error of approximation = 0.034, standardized root mean residuals = 0.03), and internal consistency (α = 0.82). Central aspects of pain was significantly associated with pain (0.50 ≤ β ≤ 0.57) but not QST. Central aspects of pain explained 33% of pain variance, rising to 42% with inflammation, age, sex, and body mass index. Central Sensitization Inventory-9 correlated with pain, not QST and explained less pain variance than CAP.

**Conclusion:**

Central aspects of pain is reliable and valid for use with people with RA and explains RA pain variance better than inflammation or CSI-9.

## Abstract 2

Supplemental Digital Content is Available in the Text.

The central aspects of pain might make important contributions to pain over and above inflammation, even in people with clinically active rheumatoid arthritis.

## 1. Introduction

Rheumatoid arthritis (RA) is a chronic inflammatory polyarthritis affecting approximately 17.6 million people globally.^6^ Joint inflammation characteristic of RA activates nociceptors, resulting in nociceptive pain. Despite advances in treatments, 40% of individuals with RA continue to experience significant pain^40^ suggestive of mechanisms beyond inflammation that contribute to the pain experience.

Nociplastic pain describes pain that is neither nociceptive nor neuropathic. Defined as “pain that arises from altered nociception despite no clear evidence of actual or threatened tissue damage causing the activation of peripheral nociceptive or evidence for disease or lesions of the somatosensory sensory system causing pain.”^27^ Nociplastic pain is common in RA and may occasionally meet the diagnostic criteria for secondary fibromyalgia^11^; however, many cases may remain undetected. Key symptoms of nociplastic pain include widespread pain disproportionate to the disease activity, fatigue, sleep disturbances, cognitive dysfunction, depression and anxiety,^18^ and pain hypersensitivity.^27^

The central aspects of pain (CAP)-knee questionnaire was initially developed to measure nociplasticity in individuals with knee pain.^2–4^ It includes 8 items addressing key symptoms of nociplastic pain, such as fatigue, anxiety, depression, pain distribution, pain-related worrying, sleep disturbance, neuropathic-like pain, and cognitive impact. Each item is associated with quantitative sensory testing (QST) evidence of pain hypersensitivity.^2^ Central aspects of pain-knee demonstrated good internal validity measuring a unidimensional construct associated with pain severity, persistent pain, and more closely correlated with pain hypersensitivity than any single related characteristic.^2–4^

Nociplastic pain is prevalent across chronic pain conditions; therefore, the CAP questionnaire was generalised and validated for people with osteoarthritis, low back pain, or fibromyalgia.^22^ Other questionnaires address constructs related to nociplastic pain, including the Generalised Pain Questionnaire^39^ emphasising pain hypersensitivity, and the Central Sensitization Inventory short-form (CSI-9)^37^ focuses on the psychometric symptoms and emotional distress not adequately explained by medical pathology.^34^ The CAP questionnaire was designed to assess both through items associated with key symptoms which were associated with evidence of hypersensitivity, making it a potentially valuable tool for measuring nociplastic pain and supporting clinical decision-making by identifying pain mechanisms beyond inflammation in RA.

Rheumatoid arthritis differs significantly from osteoarthritis and fibromyalgia in its polyarticular inflammatory pathology,^46^ relatively intensive treatment regimens, and comorbidities. Thus, the validity of tools such as the CAP questionnaire in RA cannot be assumed. This study aimed to (1) assess the validity and reliability of CAP in RA, (2) evaluate CAP's contribution to pain severity beyond inflammation in RA, and (3) investigate the CAP utility as a measure of nociplasticity, particularly its ability to capture pain hypersensitivity.

## 2. Methods

### 2.1. Participants and study design

This observational study included adults (18 years and older) with rheumatologist-diagnosed RA. To enhance real-world generalisability, participants with comorbid conditions such as fibromyalgia and degenerative joint disease were not excluded. Eligibility required self-report pain levels ≥ 3 on a numerical rating scale (NRS; 0 = no pain, 10 = worst pain imaginable) on most days in the past month to ensure clinically relevant pain. Individuals unable to provide consent or with medical or mental health conditions precluding participation were excluded.

Participants were recruited from Sherwood Forest NHS Foundation Trust (SFH), Nottingham University Hospital Trusts clinic lists, and the Investigating Musculoskeletal Health and Wellbeing cohort (IMH&W),^24^ which include individuals with RA who consented to research contact and reported disease-modifying antirheumatic (DMARDs) use. Owing to COVID-19 disruptions, some participants completed questionnaires only to validate the CAP questionnaire. Additional baseline data were sourced from Kings College London (PUMIA, RT-ILIA^36^) and Cardiff University (SOCRATES) studies. All data provided were observational, and no interventions had been performed before the point of data collection in these studies.

All participants completed the CAP questionnaire and the pain NRS. A subgroup underwent a study visit consisting of detailed assessments and questionnaires at SFH, including disease activity evaluation, QST, ultrasound imaging, and additional questionnaires. Participants repeated the CAP questionnaire to assess test–retest reliability.

Written informed consent was obtained for all participants completing study visits (detailed assessments and questionnaires). The questionnaire-only participants also returned a signed consent form. Implied consent was taken when completed questionnaires were returned without a signed consent form. The study was approved by the North of Scotland Research Ethics Committee (20/NS/0036) and the Health Research Authority and conducted in accordance with the Declaration of Helsinki. Reporting following the STROBE guidelines and the registered protocol (clinicaltrials.gov: NCT04515589).^15^

### 2.2. Patient and public involvement

The development and validation of the CAP-knee questionnaire involved extensive collaboration with individuals experiencing pain, arthritis, and members of the public. To deliberately broaden its applicability to musculoskeletal conditions, including people with RA, minor adjustments were made—replacing “knee” with “joint”—through consultations with individuals with RA to ensure clarity and inclusivity.

People with RA played a central role in the study's conceptualisation and design, ensuring relevant and meaningful outcomes. They also contributed to restructuring the study visits to enhance participant experience and reduce burden. In addition, individuals with RA served on the CAP-RA steering committee, overseeing the study management and monitoring.

Individuals with RA are coapplicants on this article and related publications. They also helped create lay summaries for dissemination, ensuring accessible information for individuals with RA and the broader public.

### 2.3. Central aspects of pain and pain questionnaire (all participants)

Participants with joint pain completed the CAP questionnaire, modified from “knee”^2–4^ to “joint.”^22^ Pain was assessed using an NRS for pain now, average, and strongest pain over the past 4 weeks.

### 2.4. Additional questionnaires (study visit and questionnaire only)

A battery of questionnaires assessed nociplastic traits and related factors including:

(1) Central Sensitization Inventory short form (CSI-9; α = 0.89)^34^ as a comparison with CAP.
(2) Modified painDETECT (α = 0.80)^33^ assess neuropathic-like pain (excluding waveform and pain intensity items).
(3) Pain-related psychological hypervigilance factors:
  (1) Pain catastrophising scale (PCS; α = 0.95).^29^
  (2) Bristol RA Fatigue Scale (BRAF; α = 0.93).^14^
  (3) Hospital Anxiety and Depression (HADS; α = 0.83 anxiety, α = 0.82 depression).^5^
  (4) Athens Insomnia Scale (ASC; α = 0.89).^38^
  (5) Cognitive Failures Questionnaire (CFQ; α = 0.92).^7^
(4) Comorbidities and functional assessments
  (1) Fibromyalgia was identified using the American College of Rheumatology (ACR) criteria (Widespread Pain Index [WPI] and Symptom Severity Score [SSS]).^41^
  (2) Physical activity assessed using the short International Physical Activity Questionnaire (IPAQ).^8^
  (3) Disability (Health Assessment Questionnaire (HAQ; α = 0.83)).^42^
  (4) Comorbidities were evaluated using the Rheumatic Disease Co-Morbidity Index,^9^ and detailed medication history.

### 2.5. Assessment of disease activity (study visit)

Disease activity was assessed using Disease Activity Score-28 (DAS28)^32^ with blood samples analysed through standard hospital procedures to calculate DAS-CRP based on C-reactive protein (CRP) levels. Tender-swollen difference (T-S_diff_)^23^ was calculated as:

(1) $${T-S}_{diff}=TJC-SJC$$

Individuals were classified as having RA if they met the American College of Rheumatology (ACR) and European League Against Rheumatism (EULAR) criteria (≥6/10).^19^

### 2.6. Assessment of pain sensitivity (study visit)

Pain sensitivity was assessed using QST, including “static” (pressure pain detection threshold [PPT]) and “dynamic” (temporal summation of pain [TSP], conditioned pain modulation [CPM]) modalities.^30^ Testing followed standardised protocols and training by 2 assessors (S.L.S. and V.G.), and participants had their eyes closed during assessments. Quantitative sensory testing was assessed at the tibialis anterior and contralateral brachioradialis (reflecting central pain processing)^12^ and medial joint line of the most painful knee (or dominant knee if pain was equal, reflecting both peripheral and central pain).^10^

#### 2.6.1. Pressure pain detection threshold

A handheld digital pressure algometer (Medoc-Algomed Advanced Medical Systems, Ramat Yishai, Israel) with a 1-cm probe applied pressure at a constant rate (50 kPa/s).^35^ Participants pressed a button when pressure changed to a sensation of pain. Pressure pain detection threshold was the mean of 3 replicate measures per site. Lower PPT values indicate greater sensitivity.

#### 2.6.2. Temporal summation of pain

Temporal summation of pain was assessed using a 256-mN punctate stimulator (MRC Systems GmbH, Heidelberg, Germany) over the quadriceps tendon of the test knee defined above. A single stimulus was applied, followed by 10 repeated stimuli at a rate of 1/s.^35^ Participants rated pain or sharpness on a visual analog scale (VAS; 0 = no pain or sharpness and 10 = worst pain or sharpness) after the single and repeated stimuli. An average of 10 was used to include the most painful stimuli, as they do not always occur as the 10th stimuli.^20^ Temporal summation of pain wind-up difference (TSP^WUD^) was calculated as:

(2) $${TSP}^{WUD}=average\;of\;10\;stimuli-single\;stimulus$$

The average of 2 TSP^WUD^ were calculated for analysis. Higher positive TSP indicate greater sensitivity.

#### 2.6.3. Conditioned pain modulation

Conditioned pain modulation was assessed by comparing PPT before and after a conditioning stimulus. The baseline (unconditioned) PPT (PPT^Mean^) was the average of 3 trials at the tibialis anterior. The conditioning stimulus was contralateral ischemic pain, induced by inflating a 15-cm blood pressure cuff on the contralateral forearm to occlude arterial blood flow. Participants squeezed a stress ball until they rated their pain at 4/10 on an NRS (0 = no pain, 10 = worst pain imaginable).^45^ Pressure pain detection threshold was then reassessed at the tibialis anterior, and the pressure cuff was immediately released. Conditioned pain modulation was calculated using Equation 3.^44,45^ A lower positive or negative CPM indicated higher sensitivity.^44^

(3) $$CPM={PPT}^{Con}-{PPT}^{Mean}$$

### 2.7. Sample size

The sample size was calculated to assess the structural validity of the CAP questionnaire using Rasch measurement theory (RMT). Assuming item calibrations are within ± 1/2 logit from stable values, with a 99% confidence interval, an optimal sample size ranged from 108 to 243, with best to poor targeting.

It was anticipated that 200 of 250 participants would complete the study, aligning with Consensus-based Standards for the selection of health Measurement Instruments (COSMIN) recommendations for RMT Analysis.^25^

### 2.8. Statistical analysis

Normality was assessed using Shapiro–Wilk, skewness, and kurtosis. All data were presented as mean ± standard deviation (SD), median and interquartile range (IQR), or frequencies, as appropriate.

Central aspects of pain was scored on a 0 to 16 continuous scale. Items 1 to 7 were scored 0 (never), 1 (sometimes), and 2 (often/always), with item 7 reverse-scored.^3^ Item 8 (body manikin) was scored based on the number of painful sites (≤ 9 sites = 0, ≥ 10 sites = 2).^22^ If 1 CAP item was missing, the score was imputed using the mean of the remaining 7 items. Central aspects of pain scores with > 1 missing item were included for RMT but excluded from additional analysis.

Floor and ceiling effects for CAP and CSI-9 were considered present if > 15% of respondents achieved the lowest/highest possible score^43^ and was deemed acceptable if < 10% of missing data.

#### 2.8.1. Validity and reliability

##### 2.8.1.1. Construct validity

Person's correlation tested the hypothesis that all CAP items were associate (r ≥ 0.3). Associated strengthens were categorised as: zero (0 – 0.25), fair (0.25 – 0.50), moderate to good (0.50 – 0.7), and good to excellent (> 0.75).^31^

##### 2.8.1.2. Structural validity

###### 2.8.1.2.1. Confirmatory factor analysis

Confirmatory factor analysis (CFA) tested if all CAP items fit a single-factor model, consistent with previous versions,^3,22^ using robust maximum likelihood CFA in R (*Lavaan* package). Model fit was assessed using COSMIN criteria.^26^ Good fit was indicated by comparative fit index (CFI)/Tucker–Lewis index (TLI) > 0.95, root mean square error of approximation (RMSEA) < 0.06, standardized root mean residuals (SRMR) < 0.08, and standardised factor loadings ≥0.3.

###### 2.8.1.2.2. Rasch measurement theory

Rasch measurement theory analysis, performed in R (*TAM* package), evaluated CAP's suitability for RA populations. Assumptions of unidimensionality (Smith procedure < 5%), local independence (Q3 residual correlations < average residual correlation + 0.2), monotonicity, and targeting (visual inspection of graphs) were assessed using COSMIN criteria.^26^

A Partial Credit Model (PCM) was used due to varying item categories. Item fit was assessed by infit and outfit Residual Mean Square (MNSQ; acceptable: 0.5 and 1.5, overfitting [too predictable or redundant] < 0.5, underfitting [item not measuring construct] > 1.5).^26^ The person Separation Index (PS ≥ 0.70) indicated acceptable internal consistency.^26^ Local invariance was assessed using Differential Item Functioning (DIF) for sex, age (< 65, ≥ 65 years), and site (Nottingham, Cardiff, London) due to different recruitment approaches, based on Nagelkerke R-squared (negligible: χ^2^ insignificant or R^2^ < 0.035, moderate; χ^2^ significant and R^2^ 0.035 – 0.07, large: χ^2^ significant and R^2^ ≥ 0.07).^17^ Alternative scoring modalities were explored for disordered response thresholds.

#### 2.8.2. Reliability and internal consistency

Cronbach alpha ≥ 0.70^26^ assessed internal consistency.

Test–retest reliability was determined using intraclass correlation coefficient (ICC_(3,1)_), calculated between days 7 and 28, to minimise recall bias. Items 1 to 7 ask about the past 7 days, and item 8 (manikin) asks about the past 4 weeks. Interobserver reliability was assessed (ICC_(2,1)_) for DAS28 and QST between independent observers (S.S., V.G.). Intraclass correlation coefficient ≥ 0.7 is deemed acceptable.^26^

#### 2.8.3. Associations with pain and central aspects of pain traits

Pearson's correlations assessed CAP-pain relationships. Bivariate and multivariable linear regression evaluated the contributions of inflammation (peripheral), CAP, CSI-9, and QST to pain, adjusted for age, sex, and body mass index (BMI). To assess CAP's relationship with psychological hypervigilance, a 7-item CAP (excluding the hypervigilance-related item) was used.

Analysis were conducted in RStudio (rstudio.com) using *psych*, *ggplot2*, *epiR, irr, summarytools, mirt, and dplyr.* Alpha = 0.5, and Benjamini and Hochberg corrected for multiple comparisons.

## 3. Results

### 3.1. Demographics

**Figure 1.**
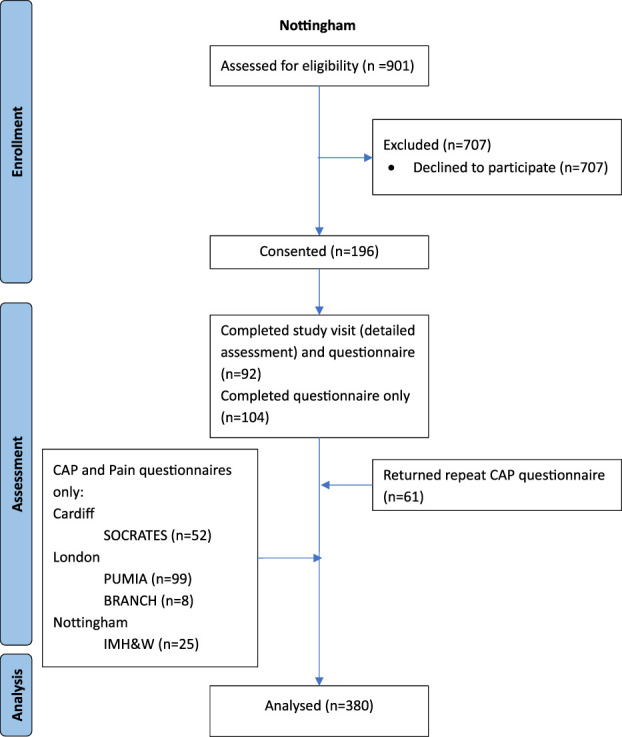
CONSORT diagram to illustrate the flow of participants through the study. SOCRATES, Patient-reported outcome measures for rheumatoid arthritis symptom severity: development of a computer adaptive test from an item bank using Rasch measurement theory; PUMIA, Phenotypes and their Underlying Mechanisms in Inflammatory Arthritis; IMH&W, Investigating Musculoskeletal Health and Wellbeing.

A total of 380 adults with rheumatologist's-diagnosed RA were recruited from Nottinghamshire (n = 221: 92 had study visits consisting of detailed assessments and questionnaires, 104 questionnaires only, 25 CAP and pain only, Fig. 1), London (n = 107) and Cardiff (n = 52) CAP and pain only. Baseline characteristics are presented in Table 1. The median (IQR) pain scores were pain now: 5/10 (3–7); strongest pain (past 4 weeks): 8 (5–9); and average pain (past 4 weeks): 6 (5–8); CAP scores: 9 (6–11); and CSI-9 scores: 21 (17–26). There was no difference in pain or demographics between recruitment sites.

**Table 1:** Participant demographics and clinical characteristics for all participants and subgroups based on location and contribution to detailed clinical assessment.

Variables	Total	Nottingham			Cardiff Questionnaire only	London Questionnaire only
		Total	Questionnaire only	Study visit		
No. of participants	380	221	129	92	52	107
Age (y)	63 (54–72)	67 (59–74)	68 (60–76)	65 (59–71)	61 (53–71)	55 (42–63)
Female sex, n (%)	281 (74%)	161 (73%)	92 (71%)	72 (78%)	40 (77%)	80 (75%)
BMI (kg/m^2^)	28.8 (25.4–32.1)	28.8 (25.4–32.1)	—	28.8 (25.4–32.1)	—	—
Comorbidities					—	—
Kidney disease	10 (3%)	10 (5%)	5 (5%)	5 (5%)		
Hypertension	59 (16%)	59 (27%)	37 (36%)	22 (24%)		
Fracture	39 (10%)	39 (18%)	17 (16%)	22 (24%)		
Heart attack	55 (14%)	55 (25%)	29 (28%)	26 (29%)		
Depression	23 (6%)	23 (11%)	13 (13%)	10 (11%)		
Diabetes	24 (6%)	24 (11%)	13 (13%)	11 (12%)		
Lung disease	15 (4%)	15 (7%)	10 (10%)	5 (5%)		
Other CVD or stroke	18 (5%)	18 (9%)	11 (11%)	7 (8%)		
Ulcer/stomach	87 (23%)	87 (39%)	48 (46%)	39 (43%)		
Other	87 (23%)	87 (39%)	48 (37%)	39 (43%)		
Comorbidity count	2 (1–3)	2 (1–2)	2 (1–4)	2 (1–3)	—	—
Current DMARDS*			—		—	—
Methotrexate	53 (14%)	53 (24%)		53 (58%)		
Sulfasalazine	20 (5%)	20 (9%)		20 (22%)		
Leflunomide	2 (1%)	2 (1%)		2 (2%)		
Etanercept	3 (1%)	3 (1%)		3 (3%)		
Rituximab	1 (0%)	1 (0%)		1 (1%)		
Adalimumab	14 (4%)	14 (6%)		14 (15%)		
Hydroxychloroquine	6 (2%)	6 (3%)		6 (7%)		
Abatacept	3 (1%)	3 (1%)		3 (3%)		
JAK inhibitor	8 (2%)	8 (4%)		8 (9%)		
Sarilumab	1 (0%)	1 (0%)		1 (1%)		
None	0	0		0		
Clinical characteristics						
Rheumatologist diagnosis	380 (100%)	221 (100%)	129 (100%)	92 (100%)	82 (100%)	107 (100%)
ACR EULAR criteria	84 (22%)	84 (65%)	—	84 (92%)	—	—
DAS28			—		—	—
TJC	11 (5–15)	11 (5–15)		11 (5–15)		
SJC	3 (1–6)	3 (1–6)		3 (1–6)		
T-S_diff_	6 (2–11)	6 (2–11)		6 (2–11)		
Global health	49 (30–64)	49 (30–64)		49 (30–64)		
DAS28-CRP	4.4 (1.2)†	4.4 (1.2)†		4.4 (1.2)†		
Pain mechanisms						
CAP (0–16)	9 (6–11)	9 (7–11)	10 (7–12)	8 (7–11)	7 (4–10)	9 (5–12)
CSI-9 (0–36)	21 (17–26)	21 (17–26)	23 (16–26)	20 (17–24)	—	—
Modified painDETECT (0–38)†	15 (8)	16 (8)	16 (9)	16 (7)	10 (4)	15 (8)
Self-reported pain						
Summated pain score (0–30)	18 (12–23)	20 (15–24)	20 (15–24)	20 (16–23)	10 (2–15)	18 (13–23)
Pain now (0–10)	5 (3–7)	5 (3–7)	5 (3–7)	5 (4–7)	5 (2–7)	4 (3–6)
Pain strongest [past 4 wk] (0–10)	8 (5–9)	8 (7–9)	8 (6–10)	8 (7–9)	5 (2–7)	8 (6–9)
Pain average [past 4 wk] (0–10)	6 (5–8)	6 (5–8)	6 (5–8)	7 (5–8)	8 (6–9)	6 (4–7)
Self-reported clinical characteristics						
HADs anxiety (0–21)	6 (3–9)	6 (3–9)	—	6 (3–9)	—	—
HADs depression (0–21)	7 (7–10)	7 (7–10)	—	7 (7–10)	—	—
Pain catastrophising scale (0–52)	15 (8–27)	15 (8–27)	—	15 (8–27)	—	—
Bristol RA fatigue scale (0–70)	36 (25–51)	36 (25–51)	42 (26–52)	34 (23–49)	—	—
Athens insomnia scale (0–24)†	10 (5)	10 (5)	—	10 (5)	—	—
Cognitive failure questionnaire (0–100)†	62 (18)	62 (18)	—	62 (18)	—	—
Widespread pain index (0–19)	3 (1–5)	3 (1–5)	—	3 (1–5)	—	—
Symptom severity score (0–12)†	5.4 (2.5)	5.4 (2.5)	—	5.4 (2.5)	—	—
Fibromyalgianess (0–31)†	8.5 (4.1)	8.5 (4.1)	—	8.5 (4.1)	—	—
Quantitative sensory testing						
PPT—Tibalis anterior (kPa)	236.2 (160.7–318.2)	236.2 (160.7–318.2)	—	236.2 (160.7–318.2)	—	—
PPT—Medial joint line (kPa)	221.5 (114.0–366.9)	221.5 (114.0–366.9)		221.5 (114.0–366.9)		
PPT—Brachioradialis (kPa)	148.0 (103.2–217.6)	148.0 (103.2–217.6)		148.0 (103.2–217.6)		
TSP (0–10)	1.3 (0.4–2.44)	1.3 (0.4–2.44)	—	1.3 (0.4–2.44)	—	—
CPM (kPa)	63.1 (4.3–140.5)	63.1 (4.3–140.5)	—	63.1 (4.3–140.5)	—	—

Data are reported as median (IQR).

* N = 35/92 (38%) were using more than 1 disease-modifying antirheumatic drug (combination therapy) at the time of recruitment.

† Mean (SD).

BMI, body mass index; CVD, cardiovascular disease; DMARDS, disease-modifying antirheumatic drugs; JAK, Janus kinases; DAS28, Disease Activity Score 28; TJC, tender joint count; SJC, swollen joint count; T-S_diff_, tender-swollen difference; ESR, erythrocyte sedimentation rate; CRP, C-reactive protein; HADs, hospital anxiety and depression scale; RA, rheumatoid arthritis; PPT, pressure pain threshold; TSP, temporal summation of pain; CPM, conditioned pain modulation.

Among the 92 participants who completed the study visits, 50% reported knee pain, 92% met ACR-EULAR RA classification criteria, 17% met ACR fibromyalgia criteria, and 32% reported having ≥ 1 joint replaced. The median PPT ranged from 148.0 to 236.2 kPa across anatomical sites (Table 1). Floor-ceiling effects were minimal for CAP (minimum: 1.4%, maximum: 0.8%) and CSI-9 (minimum: 0%, maximum: 0%). Completion rates of returned questionnaires were acceptable (93% CAP, 89% CSI-9), with limited missing data (> 1 item missing: 3% CAP, 1% CSI-9).

### 3.2. Validation and reliability

#### 3.2.1. Construct validity

Central aspects of pain items were weakly to moderately correlated (0.11 < r < 0.67, *P* < 0.05), except for depression-associated pain distribution items (r = 0.04, *P* = 0.43; Supplementary Table 1.1, http://links.lww.com/PR9/A323).

#### 3.2.2. Structural validity

##### 3.2.2.1. Confirmatory factor analysis

Confirmatory factor analysis demonstrates 7/8 CAP items loaded to a single factor (standardised factor loading: 0.33–0.83; Table 2), with good factor model fit (CFI = 0.99, TLI = 0.99, RMSEA = 0.04, SRMR = 0.03). The depression-associated item had low standardised factor loading (0.23). Multifactor models did not improve fit or item loading.

**Table 2:** Confirmatory factor analysis of the central aspects of pain questionnaire (n = 380).

Item label	CAP
Neuropathic -like pain	0.47
Fatigue	0.69
Cognitive impact	0.83
Pain-related worrying	0.79
Anxiety	0.51
Sleep	0.69
Depression	0.23
Pain distribution	0.33
Model fit	
RMSEA	0.04 (0.00, 0.07) *P* = 0.823
SRMR	0.03
AIC	5823.31
BIC	5920.75
CFI	0.99
TLI	0.99

CAP demonstrates good overall model fit, with the depression item demonstrating low standardised factor loading.

Item labels indicate psychological or symptom constructs that have been associated with the individual items included within the CAP questionnaire. The single items within CAP should not be taken to represent reliable measures of those constructs.

AIC, Akaike information criteria; BIC, Bayesian information criteria; CAP, central aspects of pain; CFI, comparative fit index > 0.95 indicate good fit; RMSEA, root mean square error of approximation, with (95% Confidence interval) < 0.06 reasonable fit; SRMR, Standardised Root Mean Square Residual < 0.08 good fit; TLI, Tucker–Lewis Index > 0.95 indicate good fit.

##### 3.2.2.2. Rasch measurement theory

Expected item ordering and no mistargeting (category characteristic curves, person-item maps) were demonstrated. Central aspects of pain violated assumptions of unidimensionality and local dependence (Supplementary Table 1.3, http://links.lww.com/PR9/A323). Although the summary item fit residual was good (Table 3), the depression-associated and pain distribution items demonstrated evidence of underfitting (Supplementary Figures 1.1–1.3, http://links.lww.com/PR9/A323). Principal Component Analysis confirmed all 8 items loaded onto the first component. There were no differences in difficulty except for the depression-associated item across sites (Supplementary Table 1.6–1.8, http://links.lww.com/PR9/A323) determined by DIF models.

**Table 3:** Fit statistics and summary item-person interaction statistics for central aspects of pain using the partial credit model (n = 380).

Model	χ^2^ (df)	*P*	Item fit residual Mean (SD)	Person fit residual Mean (SD)	PSI	Percentage of significance *t* test (95% CI)	Q3 correlations Average (range)
CAP	72.9 (28)	<0.001	−0.20 (2.98)	−0.03 (1.29)	0.74	11% (8.4%–15.1%)	**0.09 (−0.28 ≤ Q3 ≤ 0.31)**
Ideal values		>0.05	0 (1)	0 (1)	≥0.70	<5%	Average* + 0.2

Item label	Difficulty logit	SE logit	Outfit MNSQ	Infit MNSQ
Neuropathic -like pain	0.57	0.07	1.10	1.07
Fatigue	−1.96	0.06	0.71	0.79
Cognitive impact	−0.66	0.07	0.77	0.79
Pain-related worrying	−0.67	0.06	0.80	0.81
Anxiety	1.16	0.02	0.95	0.99
Sleep	−0.89	0.04	0.85	0.86
Depression	0.45	0.08	1.44	1.32
Pain distribution	0.41	0.09	2.03	1.37

Fatigue was the easiest item (difficulty logit −1.96), anxiety the most difficult item (difficulty logit 1.16). Neuropathic-like pain, fatigue, cognitive impact, pain-related worrying, anxiety, and sleep demonstrated good fit (MNSQ within the range of 0.7–1.3). Depression and pain distribution demonstrated underfitting. N = 380, Item labels indicate psychological or symptom constructs that have been associated with the individual items included within the CAP questionnaire. The single items within CAP should not be taken to represent reliable measures of those constructs.

* CAP: Central Aspects of Pain, χ^2^ (df): chi-squared (degrees of fredom), PSI: person separation index, MNSQ: mean square residual, SE: Standard Error. Items in bold indicate violation of assumptions

The polyarticular nature of RA might compromise the validity of the pain distribution item. Alternative scoring (CAP7 excluding pain distribution) and CAP6 (excluding pain distribution and depression-associated items) improved unidimensionality but violated with RMT assumptions (Supplementary Table 1.3, http://links.lww.com/PR9/A323). The 8-item CAP was retained for primary analyses.

#### 3.2.3. Reliability and internal consistency

Central aspects of pain demonstrated good internal consistency (Cronbach α = 0.82, n = 380) and reliability (ICC_(3,1)_ 0.86 (95% CI 0.75–0.94)).

Interobserver reliability between independent observers (S.S., V.G.) was excellent for DAS28 (DAS-CRP ICC_(2,1)_ 0.92 [95% CI 0.84–0.97]). Quantitative sensory testing reliability was acceptable to moderate (PPT medial joint line ICC_(2,1)_ 0.85 [95% CI 0.70–0.94]), tibialis anterior ICC_(2,1)_ 0.85 (95% CI 0.69–0.93), brachioradialis (ICC_(2,1)_ 0.77 [95% CI 0.49–0.90]), TSP (ICC_(2,1)_ 0.50 [95% CI 0.13–0.74]), and CPM (ICC_(2,1)_ 0.48 [95% CI 0.13–0.73]).

### 3.3. Associations of central aspects of pain with pain and linked traits

Numerical rating scale pain scores (pain now, average, and strongest pain) were highly intercorrelated (0.67 < r < 0.75) and summated as the dependent variable in the regression models. Central aspects of pain was moderately associated with pain and weakly associated with swollen joint count (SJC), remaining significant after adjustment for age, sex, and BMI (Table 4). No significant associations were shown for QST. Central aspects of pain was associated with psychological hypervigilance constructs and widespread pain, with and without adjustment for age, sex, and BMI (Table 5).

**Table 4:** Individual bivariate (unadjusted model) and multivariable (adjusted model, for age, sex, and body mass index) linear regression models of associations between central aspects of pain or central sensitization inventory short form-9 scores and pain, pain sensitivity, or inflammation.

	CAP unadjusted model			CSI-9 unadjusted model		
	β	95% CI	*P*	β	95% CI	*P*
Pain (n = 343)						
Combined pain	0.57	0.48, 0.66	<0.001	0.63	0.50, 0.76	<0.001
Pain now	0.50	0.41, 0.59	<0.001	0.51	0.37, 0.64	<0.001
Strongest pain past 4 wk	0.55	0.46, 0.64	<0.001	0.56	0.42, 0.71	<0.001
Average pain past 4 wk	0.53	0.43, 0.63	<0.001	0.59	0.47, 0.71	<0.001
Modified painDETECT	0.55	0.43, 0.67	<0.001	0.60	0.48, 0.72	<0.001
Pain sensitivity (n = 90)						
PPT medial joint line	−0.05	−0.25, 0.15	0.606	−0.08	−2.7, 0.10	0.374
PPT brachioradialis	−0.001	−0.19, 0.18	0.985	−0.02	−0.21, 0.16	0.799
PPT tibialis anterior	−0.07	−0.25, 0.12	0.460	−0.07	−0.26, 0.11	0.433
TSP	0.06	−0.13, 0.25	0.519	0.10	−0.08, 0.29	0.279
CPM	−0.04	−0.22, 0.15	0.692	0.00	−0.19, 0.19	0.989
Inflammation (n = 90)						
SJC	0.20	0.28, 0.39	0.024	0.09	−0.09, 0.28	0.312
CRP	0.12	−0.06, 0.31	0.194	0.13	−0.06, 0.32	0.179

	CAP adjusted model			CSI-9 adjusted model		
	β	95% CI	*P*	β	95% CI	*P*
Pain (n = 343)						
Combined pain	0.75	0.54, 0.96	<0.001	0.51	0.27, 0.74	<0.001
Pain now	0.52	0.31, 0.73	<0.001	0.32	0.10, 0.55	0.005
Strongest pain past 4 wk	0.69	0.47, 0.91	<0.001	0.40	0.15, 0.65	0.002
Average pain past 4 wk	0.65	0.46, 0.83	<0.001	0.52	0.32, 0.72	<0.001
Modified painDETECT	0.52	0.33, 0.70	<0.001	0.38	0.18, 0.58	<0.001
Pain sensitivity (n = 90)						
PPT medial joint line	−0.06	−0.26, 0.15	0.602	−0.09	−0.28, 0.11	0.384
PPT brachioradialis	−0.01	−0.21, 0.19	0.915	0.00	−0.19, 0.19	0.996
PPT tibialis anterior	−0.07	−0.28, 0.13	0.470	−0.05	−0.25, 0.15	0.629
TSP	0.07	−0.12, 0.27	0.459	0.11	−0.08, 0.30	0.260
CPM	−0.06	−0.25, 0.14	0.570	0.01	−0.19, 0.20	0.940
Inflammation (n = 90)						
SJC	0.23	0.04, 0.43	0.016	0.07	−0.12, 0.27	0.458
CRP	0.19	−0.01, 0.38	0.064	0.14	−0.05, 0.34	0.148

Β, Standardised beta coefficient; CAP, central aspects of pain; CPM, conditioned pain modulation; CRP, C-reactive protein; CSI-9, central sensitization inventory short form; PPT, pressure pain detection threshold; SJC, swollen joint count; TSP, temporal summation of pain; modified painDETECT, pain detect score with item 6 removed due to inclusion in CAP; 95% CI, 95% confidence interval.

**Table 5:** Associations between central aspects of pain and related characteristics.

Characteristic	Questionnaire	Unadjusted		Adjusted	
		Β (95% CI)	*P*	Β (95% CI)	*P*
Neuropathic -like pain	painDETECT	0.55 (0.45, 0.65)	<0.001	0.49 (0.30, 0.69)	<0.001
Fatigue	BRAF total fatigue	0.73 (0.64, 0.82)	<0.001	0.70 (0.56, 0.83)	<0.001
Cognitive impact	CFQ total	−0.36 (−0.52, −0.19)	<0.001	−0.38 (−0.57, −0.19)	0.001
Pain-related worrying	PCS total	0.57 (0.42, 0.71)	<0.001	0.58 (0.41, 0.74)	<0.001
Anxiety	HADs anxiety	0.43 (0.28, 0.58)	<0.001	0.43 (0.27, 0.59)	<0.001
Sleep	AIS	0.50 (0.34, 0.65)	<0.001	0.51 (0.35, 0.68)	<0.001
Depression	HADS depression	0.45 (0.31, 0.59)	<0.001	0.48 (0.32, 0.64)	<0.001
Pain distribution	WPI	0.26 (0.09, 0.43)	0.002	0.29 (0.11, 0.46)	0.001
Pain distribution	Fibromyalgianess score	0.38 (0.22, 0.23)	<0.001	0.42 (0.26, 0.58)	<0.001

Standardised beta coefficients are derived from separate multivariable linear regression models with the CAP. CAP scores were modified by omission of the item that was originally derived from a questionnaire addressing that characteristic tested by the model. For example, the CAP fatigue item was omitted from CAP scores in linear regression against BRAF fatigue scores. Models are presented unadjusted and adjusted for age, sex and BMI.

AIS, Athens insomnia scale; B, standardised beta; BRAF, Bristol rheumatoid arthritis fatigue scale, CFQ, cognitive failures questionnaire; HADs, hospital anxiety and depression scale; painDETECT, total score from modified painDETECT questionnaire; PCS, pain catastrophising scale; SE, standard error; WPI, widespread pain index (manikin score).

Inflammation (CRP and SJC) explained 10% of pain variance (Table 6). Central aspects of pain alone explained 37%. Including CAP and inflammation indices in a single model explained 38% of pain variance (28% more than inflammation indices alone), and a full model, including age, sex, BMI, SJC, CRP, and CAP score, explained 42% of pain variance (Table 6).

**Table 6:** Bivariable and multivariable models for contributions of inflammation and central aspects of pain to summated pain scores.

	B	95% CI	*P*	R^2^	*P*
Univariable models					
CRP	0.19	−0.02, 0.42	0.070	0.040	0.070
SJC	0.20	0.00, 0.43	0.049	0.047	0.0049
CAP	0.60	0.43, 0.78	<0.001	0.365	<0.001
CSI-9	0.38	0.18, 0.58	<0.001	0.143	<0.001
Model 1 (inflammation)					
CRP	0.23	0.01, 0.43	0.038		
SJC	0.24	0.03, 0.45	0.027		
	R^2^ = 0.097, *P* = 0.017				

	CAP			CSI-9		
	B	95% CI	*P*	B	95% CI	*P*
Model 2 (inflammation and CAP or CSI-9)						
CRP	0.10	−0.08, 0.28	0.283	0.17	−0.03, 0.37	0.098
SJC	0.08	−0.10, 0.27	0.376	0.20	−0.01, 0.40	0.051
CAP/CSI-9	0.56	0.38, 0.75	<0.001	0.33	0.13, 0.54	0.002
	R^2^ = 0.378, *P* < 0.001			R^2^ = 0.205, *P* < 0.001		
Model 3 (inflammation and CAP or CSI-9, fully adjusted)						
CRP	0.09	−0.09, 0.28	0.301	0.17	−0.03, 0.37	0.104
SJC	0.11	−0.07, 0.30	0.222	0.24	0.04, 0.44	0.020
CAP/CSI-9	0.58	0.39, 0.77	<0.001	0.39	0.18, 0.59	<0.001
Age	0.12	−0.06, 0.30	0.175	0.15	−0.05, 0.36	0.140
Sex	0.13	−0.05, 0.31	0.145	0.18	−0.02, 0.39	0.075
BMI	−0.09	−0.27, 0.08	0.298	−0.05	−0.25, 0.15	0.606
	R^2^ = 0.419, *P* < 0.001			R^2^ = 0.266, *P* < 0.001		

N = 83. Step-wise multivariable linear regression models explaining summated pain scores.

B, standardised beta; BMI, body mass index; CAP, central aspects of pain; CRP, C-reactive protein; CSI-9, central sensitization inventory short form; SJC, swollen joint count; 95% CI, 95% confidence interval.

Central Sensitization Inventory-9 correlated with CAP (ρ = 0.66) and demonstrated significant associations with pain but not with QST indices of central sensitisation or inflammation indices (Table 4). Central Sensitization Inventory-9 alone explained 14% of pain variance. Including CSI-9 and inflammation indices in a single model explained 21% of pain variance, 11% more than inflammation indices (CRP, SJC) alone (Table 6). A full model, including age, sex, BMI, SJC, CRP, and CSI-9, explained 27% of pain variance (Table 6). Central aspects of pain explained 15% more pain variance in fully adjusted models than CSI-9.

## 4. Discussion

Central aspects of pain may reflect psychological hypervigilance rather than nociceptive sensitivity, highlighting a potential CNS manifestation of pain in RA. It displays evidence of reliability and validity as a potential measure of psychological hypervigilance with some caveats. Central aspects of pain aligns with a unidimensional construct linked to pain severity beyond inflammation, explaining pain severity better than CSI-9. Neither CAP nor CSI-9 were significantly correlated with QST modalities selected as proxy measures of central sensitisation. Our findings suggest that CAP may, therefore, reliably measure psychological hypervigilance as a CNS manifestation of pain in inflammatory polyarthritis, indicating shared mechanisms across chronically painful musculoskeletal conditions. Although polyarticular inflammation contributes more to RA pain than central sensitisation as measured by QST, other CNS mechanisms linked to psychological hypervigilance seem to play a significant role.

### 4.1. Central aspects of pain development and feasibility

Central aspects of pain was originally developed to assess nociplastic pain contributions to chronic knee pain and adapted for broader use.^22^ Central aspects of pain proved feasible and acceptable in people with RA with low data missingness and no floor-ceiling effects. Internal consistency, CFA, and RMT supported its unidimensional structure despite some misfitting. Alternative scoring improved model fit in some respects but introduced new dependencies. No differences in difficulty (DIF) were found by age or sex, but the depression-associated item varies across geographical study sites. Nociplastic pain is shared across musculoskeletal conditions. Given alternative scoring did not improve the model fit and CAP's generalisability across musculoskeletal conditions and settings, we recommend maintaining the standard CAP scoring protocol.^22^

### 4.2. Central aspects of pain association with pain and inflammation

Pain is a complex multidimensional experience that no single measure of pain severity can adequately capture. Inflammation is the primary driver of pain in RA. Although inflammatory indices (CRP, SJC) were associated with pain, they only explained 10% of the pain variance. Central aspects of pain was strongly associated with self-reported pain, including pain now, average pain, and strongest pain over 4 weeks. Central aspects of pain alone explained 37% of pain variance. Adding CAP to inflammation in fully adjusted models increased this to 42%, suggesting that CAP may make an important contribution to pain even in active RA.

Central aspects of pain has previously been shown to be associated with QST evidence of central sensitisation in chronic knee pain,^2^ indicating that CAP may reflect both psychological hypervigilance and pain hypersensitivity. In RA, neither CAP nor CSI-9 was significantly associated with any QST modality. This aligns with previous studies suggesting that CSI reflects psychological hypervigilance, and neither CSI nor PSQ reflects nociceptive sensitivity.^1,16^ Questionnaires and QST modalities might reflect discrete aspects of CNS manifestations of pain, suggesting CNS contributions may extend beyond what QST alone can detect. Questionnaires combined with QST may provide a clearer measure of CNS manifestations of pain.

### 4.3. Comparison of central aspects of pain and central sensitization inventory-9

Central Sensitization Inventory was developed to classify conditions linked to central sensitisation (e.g., fibromyalgia).^34^ CSI-9 was refined independently of CAP development.^28^ Central aspects of pain and central sensitization inventory-9 were strongly associated, which is unsurprising given some overlap. Central aspects of pain explained a greater proportion of pain variance (37%) compared with CSI-9 (14%). When added to inflammation indices, CAP provided a more substantial increase in explained pain variance than CSI-9, suggesting possible advantages of CAP over CSI-9. Longitudinal studies are needed to clarify their comparative utility in measuring, classifying, or predicting CNS manifestations of pain.

### 4.4. Strengths and limitations

This sample broadly reflected RA demographics. Although efforts were made to promote diversity, 96% were White, limiting cross-cultural generalisability. As a cross-sectional study, causality cannot be inferred. Longitudinal interventional studies are required to determine whether CAP reflects psychological hypervigilance as a CNS manifestation that exacerbates pain. Central aspects of pain, inflammation, and demographics explain 42% of pain variance, indicating that other unmeasured factors contribute. Swollen joint count and C-reactive protein are crude inflammation markers, while no single QST protocol can definitively measure central sensitisation and is subject to measurement error and influenced by external factors (eg, psychological state, medication, and environment).^13^ Our QST protocols were designed to investigate central pain processing in people with musculoskeletal disease. They use mechanical stimuli and stimulate deep rather than cutaneous tissues. Alternative protocols might reveal different associations, for example, protocols designed to assess sensory deficits and abnormal sensitivity characteristic of neuropathy.^21^ Quantitative sensory testing was undertaken following standardised training and protocols, conducted at a single site in a quiet room. Pressure pain detection threshold reliability was acceptable, but TSP and CPM were below acceptable levels. Quantitative sensory testing assessments were separated by > 5 minutes; however, some residual effects may have been present. Poor reliability may cause greater variability, obscuring any real association between TS or CPM with CAP or CSI-9.

Although CAP provides a continuous measure of nociplasticity, threshold scores for pathology, prognosis, or treatment response remain undefined. Central aspects of pain is not targeted at pain-free individuals. Including non-RA controls would allow disease-specific factors to be significantly different between groups. In an attempt to control for this the wide range of RA phenotypes within our study population was assessed. Ensuring the focus was on pain mechanisms rather than disease vs control.

## 5. Conclusion

Central aspects of pain demonstrates strong construct validity and internal consistency in RA, measuring a CNS-driven construct potentially linked to psychological hypervigilance. Its validation aligns with prior work in knee pain, supporting its applicability across inflammatory and noninflammatory musculoskeletal conditions. Future research should explore CAP's role in predicting pain prognosis and treatment response.

## Disclosures

E.C. has received research grants, consultancy, and speaker honoraria from Abbvie, Bio-Cancer, Biocon, Biogen, Bristol Myer Squibbs, Chugai Pharma, Eli Lilly, Fresenius Kabi, Galapagos, Gilead, Inmedix, Janssen, Novartis, Pfizer, Sanofi, UCB, and Viatris. B.K. has received research grants, consultancy, and speaker honoraria from Abbvie, Eli Lilly, UCB, Galapagos, Janssen, Novartis, and Pfizer. D.A.W. received research grants, consultancy, and speaker honoraria from Pfizer Ltd, UCB Pharma, Orion Corporation, GlaxtonSmithKline Research and Development, Eli Lilly Company, Grunenthal GmbH, Contura International A/S, AbbVie Inc, and Medscape International. D.F.M. has grant support from Eli Lilly, Pfizer, and UCB. Others: no interest to declare.

## Appendix A. Supplemental digital content

Supplemental digital content associated with this article can be found online at http://links.lww.com/PR9/A323.

## Supplementary Material

SUPPLEMENTARY MATERIAL
