# Supplementary material for: Validity and contributions to pain from the central aspects of pain questionnaire in rheumatoid arthritis
Source: Pain Rep. 2025 Jun 20;10(4):e1295. doi: 10.1097/PR9.0000000000001295 (PMC12185087; doi:10.1097/PR9.0000000000001295)
Supplement: SUPPLEMENTARY MATERIAL [file painreports-10-e1295-s001.docx]

# **Supplementary Material 1.**

## Hypothesis testing for construct validity.

CAP items were correlated with each other (Supplementary Table 1.1). Items 1 to 6 correlated with magnitudes r>0.3. Items relating to depression-associated and pain distribution had magnitudes <0.3.

| **Supplementary Table 1.1.** Pearson’s Correlation Coefficients between individual Central Aspect of Pain questionnaire items. | | | | | | | | |
| --- | --- | --- | --- | --- | --- | --- | --- | --- |
| **Item label** | **(1)** | **(2)** | **(3)** | **(4)** | **(5)** | **(6)** | **(7)** | **(8)** |
| **(1) Neuropathic like pain** |  | 0.33 | 0.34 | 0.35 | 0.26 | 0.39 | 0.11 | 0.18 |
| **(2) Fatigue** |  |  | 0.56 | 0.52 | 0.35 | 0.49 | 0.15 | 0.29 |
| **(3) Cognitive impact** |  |  |  | 0.67 | 0.40 | 0.59 | 0.19 | 0.25 |
| **(4) Catastrophising** |  |  |  |  | 0.44 | 0.51 | 0.15 | 0.23 |
| **(5) Anxiety** |  |  |  |  |  | 0.29 | 0.16 | 0.20 |
| **(6) Sleep** |  |  |  |  |  |  | 0.17 | 0.24 |
| **(7) Depression** |  |  |  |  |  |  |  | 0.04 |
| **(8) Pain distribution** |  |  |  |  |  |  |  |  |
| All items display associations with each other expect items of depression-associated and pain distribution. Data are presented as Pearsons’ correlation coefficients. Item labels indicate psychological or symptom constructs that have been associated with the individual items included within the CAP questionnaire. The single items within CAP should not be taken to represent reliable measures of those constructs. | | | | | | | | |

## Structural Validity

### *Confirmatory Factor Analysis*

Confirmatory Factor Analysis (CFA) demonstrated that all items were loaded to a single factor, and standardised factor loadings were >0.3 (range 0.32 to 0.59) for 7/8 items, with the depression-associated item loading at 0.16 (Supplementary Table 1.2). The pain manikin also has a low loading at 0.32.

Rheumatoid Arthritis is a polyarticular disease with inflammation causing pain in multiple sites, which may explain the low manikin factor loading. The depression-associated item is the only item which is reverse-scored. Additional CFA models were undertaken using modified CAP: 1) CAP7: with the pain manikin removed (justified by RA being a polyarticular disease), 2) CAP6: with the pain manikin and depression-associated items removed (justified by possible poor fitting). 3) CAP with local dependence included in the CFA model. Removing the pain manikin item, or including local dependence did not alter the overall model fit; removing the pain manikin and depression-associated items gave RMSEA =0.06 (Supplementary Table 1.2)

| **Supplementary Table 1.2.** Confirmatory factor analysis of the CAP questionnaire. | | | | |
| --- | --- | --- | --- | --- |
|  | **Central Aspects of Pain** | | | |
| **Item label** | **CAP** | **CAP7** | **CAP6** | **CAP including local dependence** |
| Neuropathic -like pain | 0.47 | 0.46 | 0.46 | 0.48 |
| Fatigue | 0.69 | 0.68 | 0.68 | 0.69 |
| Cognitive impact | 0.83 | 0.83 | 0.52 | 0.78 |
| Catastrophising | 0.79 | 0.79 | 0.79 | 0.75 |
| Anxiety | 0.51 | 0.50 | 0.50 | 0.50 |
| Sleep | 0.69 | 0.67 | 0.69 | 0.70 |
| Depression | 0.23 | 0.23 | - | 0.22 |
| Pain distribution | 0.33 | - | - | 0.34 |
| Cognitive impact ~ Catastrophising | - | - | - | 0.18 |
| **Model Fit** |  |  |  |  |
| RMSEA | 0.04 (0.00, 0.07) P=0.823 | 0.04 (0.00, 0.08) P=0.618 | 0.06 (0.01, 0.10) P=0.344 | 0.03 (0.01, 0.04) P=0.858 |
| SRMR | 0.03 | 0.03 | 0.03 | 0.03 |
| AIC | 5823.31 | 4712.56 | 3951.74 | 5635.22 |
| BIC | 5920.75 | 4767.19 | 3998.57 | 5647.44 |
| CFI | 0.99 | 0.99 | 0.99 | 0.99 |
| TLI | 0.99 | 0.98 | 0.98 | 0.99 |
| CAP demonstrates good overall model fit, with the depression-associated item demonstrating low standardised factor loading. Removing the pain distribution item does not alter the model fit. Removing the pain distribution and depression-associated items RMSEA indicates the model fit is no longer acceptable.  **AIC:** Akaike information criteria; **BIC:** Bayesian information criteria; **CAP:** Central Aspects of Pain, **CAP7L** Central Aspects of Pain minus pain distribution item; **CAP6:** Central Aspects of Pain minus pain distribution and depression items; **CFI:** comparative fit index >0.95 indicate good fit; **RMSEA:** root mean square error of approximation, with (95% Confidence interval) <0.06 reasonable fit; **SRMR:** Standardised Root Mean Square Residual <0.08 good fit; **TLI:** Tucker-Lewis Index >0.95 indicate good fit. Item labels indicate psychological or symptom constructs that have been associated with the individual items included within the CAP questionnaire. The single items within CAP should not be taken to represent reliable measures of those constructs. | | | | |

### *Rasch Measurement Theory (RMT)*

CAP violated the local dependence and unidimensionality assumptions based on the COMIN criteria (Supplementary Table 1.3). Local independence was violated (between items 3-4, 3-6). Category characteristic curves and person-item maps displayed expected category ordering distributions across items for CAP (Supplementary Figures 1.1 - 1.2) with deviations in the observed fit from the expected fit for depression-associated and pain distribution items (Supplementary Figure 1.3). The person ability extends over the majority of item difficulty ranges with the exception of pain distribution, with most person abilities sitting at the locations of the item difficulties, indicating no mistargeting (Supplementary Figure 1.2). The summary item-fit residual statistics indicate some misfits (Supplementary Table 1.3). The person-item fit was good, but items related to depression and pain distribution displayed some evidence of underfitting (Supplementary Table 1.4). Principal Component Analysis of the residuals found that all 8 items loaded on the first component. Differential Item Functioning (DIF) demonstrated no difference in sex (male, female, Supplementary Table 1.5), age (<66, ≥65 years, Supplementary Table 1.6), and geographical site (Nottingham, Cardiff, London, Supplementary Table 1.7), with the exception of depression-associated item across sites.

Alternative scoring was explored by removing the pain distribution item due to the polyarticular nature of rheumatoid arthritis (CAP7) and removing the pain distribution and depression-associated items based on the CAP CFA and RMT results (CAP6). CAP7 violated the assumptions of local independence (Supplementary Table 1.3) between items 3-4, whilst meeting the threshold for unidimensionality. Removing the pain distribution item resulted in greater deviations from the ideal item residual values (Supplementary Table 1.3). CAP6 met the assumption of unidimensionality based on the COMIN criteria but violated the assumption of local independence (Supplementary Table 1.3) between items 3-4 and 3-5 and resulted in a model fit no better than removing the pain distribution item (CAP7).

Although CAP has some slight misfitting, CAP7 and CAP6 also violate the assumptions of RMT. They improve some components of the model fit whilst decreasing others. Based on the theoretical underpinnings in the development of CAP, RMT results, and our primary aim to determine whether CAP can be used in people with RA, rather than creating an optimal questionnaire for people with RA, CAP was taken forward for all additional analysis. CAP may provide optimal fit, but users should be cautious about the potential underfitting of depression-associated and pain distribution items.

| **Supplementary Table 1.3.** Summary item-person interaction statistics | | | | | | |  |
| --- | --- | --- | --- | --- | --- | --- | --- |
| **Model** | **χ^2^ (df)** | **p-value** | **Item fit residual**  Mean (SD) | **Person fit residual**  Mean (SD) | **PSI** | **Percentage of significance t-test** (95% CI) | **Q3 correlations** average (range) |
| CAP | 72.9 (28) | **<0.001** | -0.20 (2.98) | -0.03 (1.29) | 0.74 | **11.4% (8.4% to 15.1%)** | **0.09 (-0.28≤Q3≤0.31)** |
| CAP7 | 75.1 (21) | **<0.001** | -0.32 (1.52) | -0.02 (1.5) | 0.73 | 1.1% (0.3% to 2.7%) | **-0.11 (-0.34≤Q3≤0.25)** |
| CAP6 | 35.7 (15) | **<0.001** | -0.52 (1.83) | -0.05 (1.86) | 0.72 | 2.7% (1.3% to 4.9%) | **-0.15 (-0.35≤Q3≤0.13)** |
| Ideal Values |  | >0.05 | 0 (1) | 0(1) | ≥0.70 | <5% | Average+0.2 |
| CAP displays some item misfits, violations of unidimensionality and local independence. Removing the pain distribution (CAP7) item improves the unidemensionality but not the item fit residual. Removing the pain distribution and depression-associated items (CAP6) does not improve the fit beyond removing the pain distribution item alone.  **χ^2^:** chi-squared; **df:** degrees of freedom; **SD:** standard deviation; **PSI:** Person separation Index; **95% CI:** 95% confidence interval; **CAP:** Central Aspects of Pain, **CAP7:** Central Aspects of Pain minus pain distribution item; **CAP6:** Central Aspects of Pain minus pain distribution and depression items; **Percentage of significance t-test**: measure of unidemensionality; **Q3 correlations:** measure of local independence. | | | | | | | |

|  | **CAP** |  | **CAP7** |  | **CAP6** |
| --- | --- | --- | --- | --- | --- |
| 1a. | 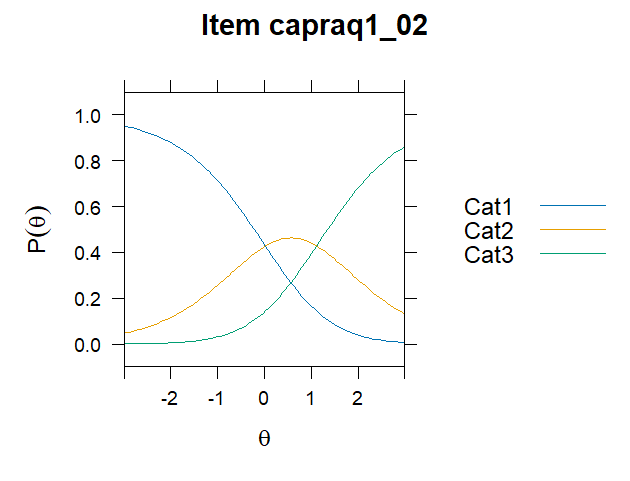 | 2a. | 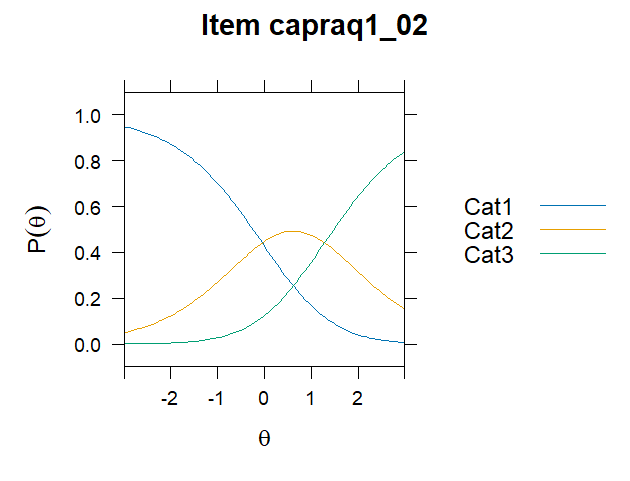 | 3a. | 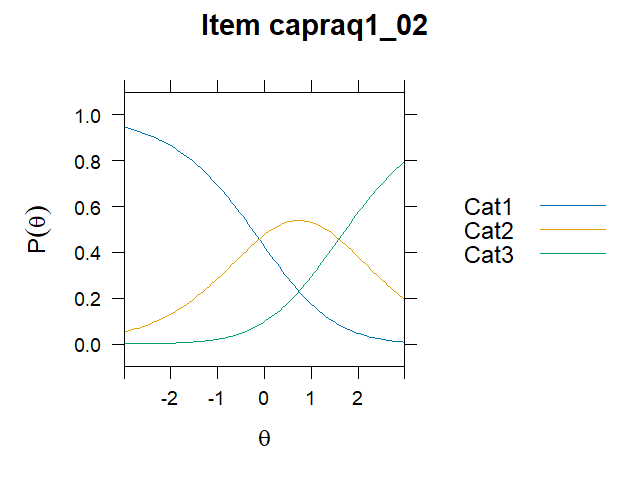 |
| 1b. | 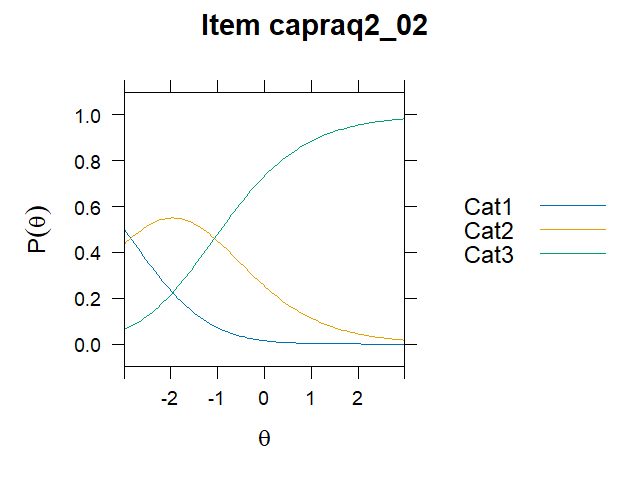 | 2b. | 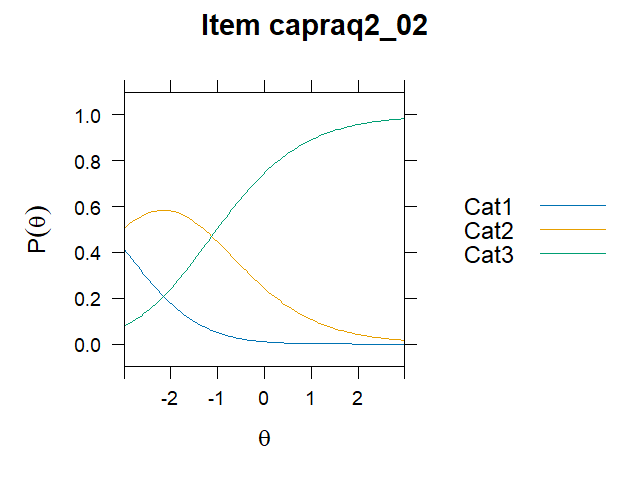 | 3b. | 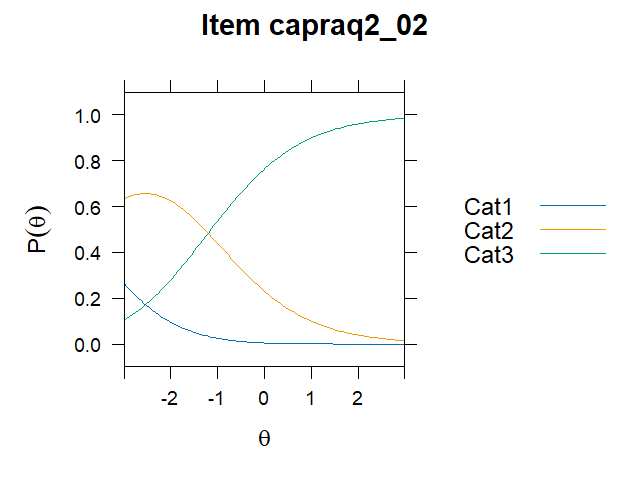 |
| 1c. | 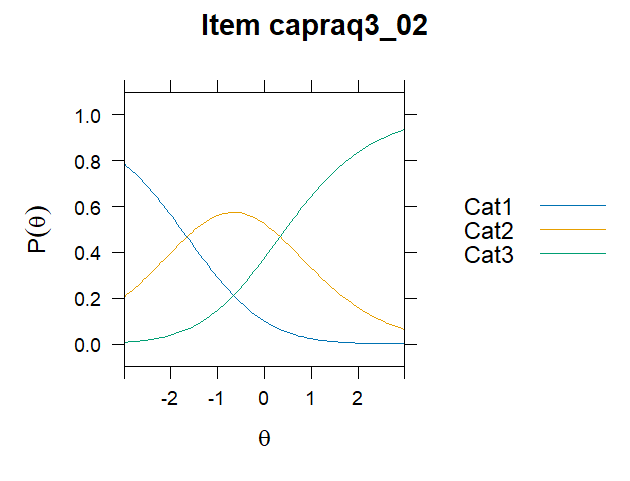 | 2c. | 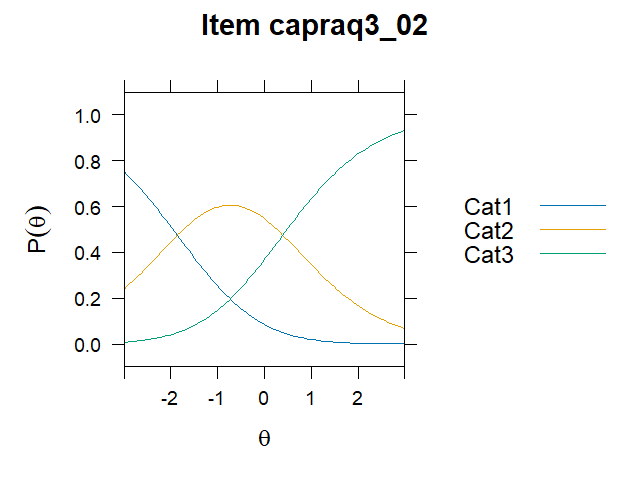 | 3c. | 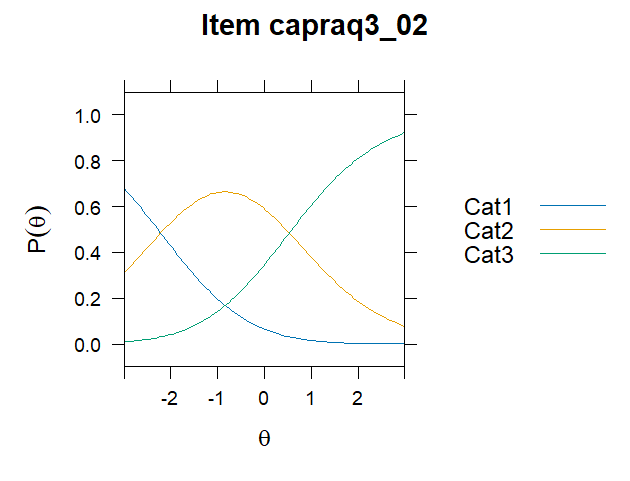 |
| 1d. | 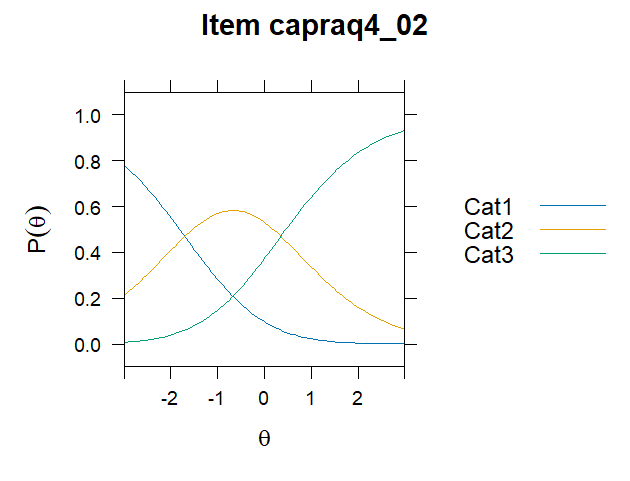 | 2d. | 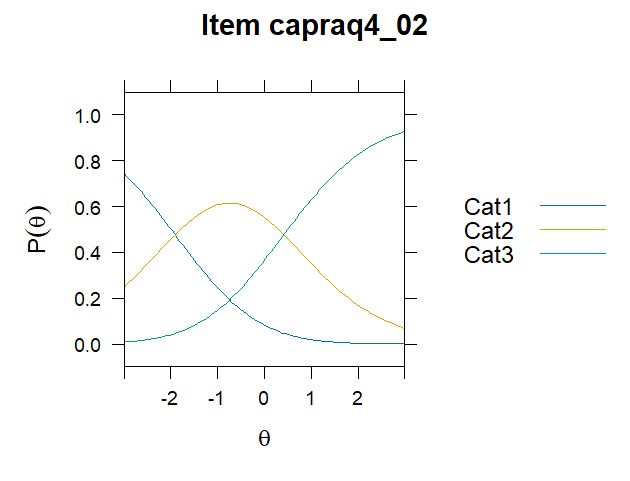 | 3d. | 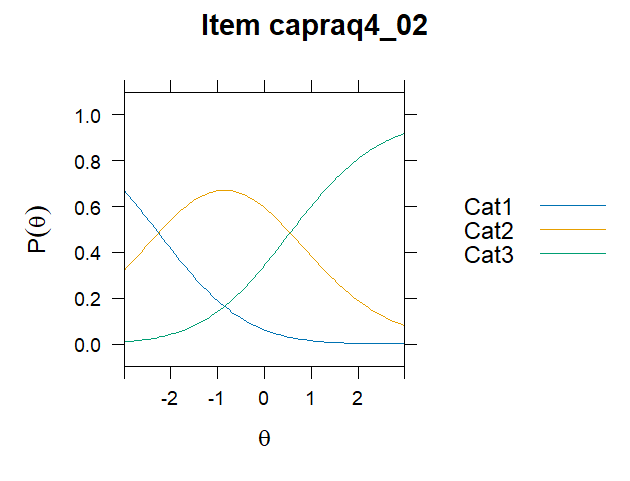 |
| 1e. | 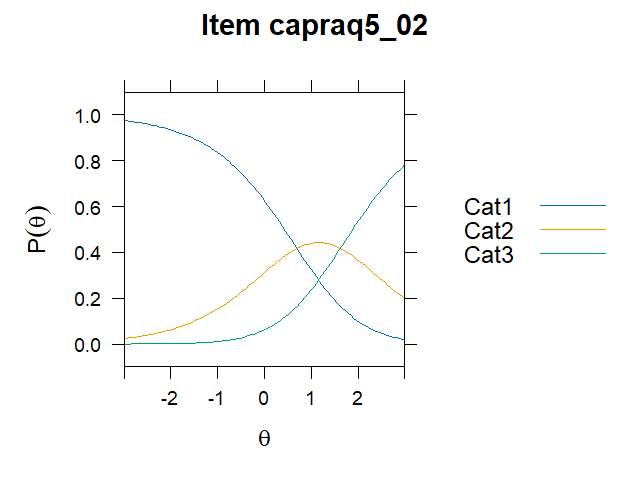 | 2e. | 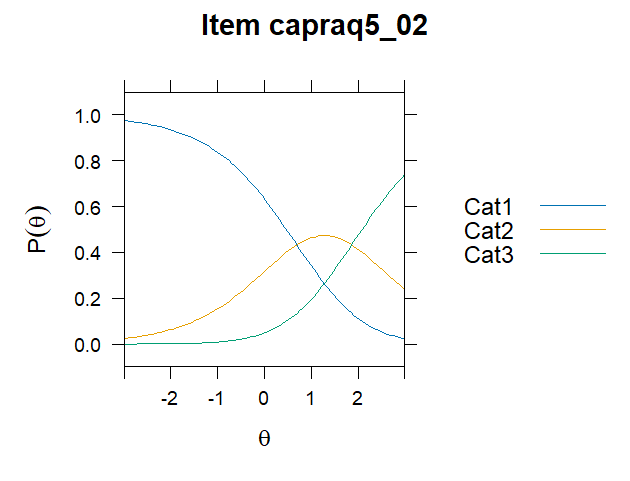 | 3e. | 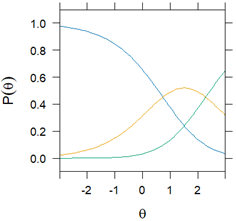 |
| 1f. | 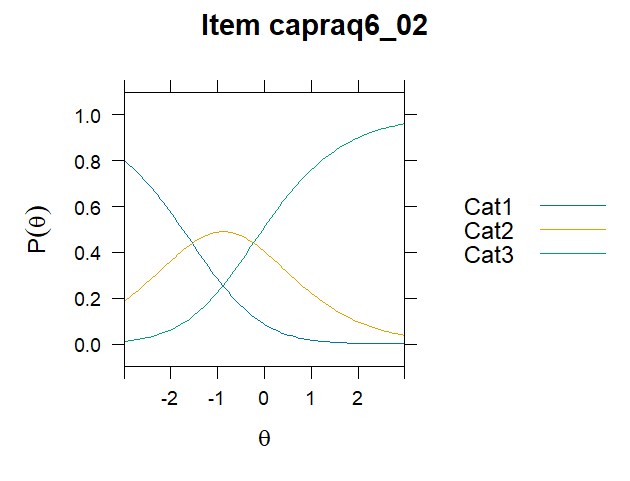 | 2f. | 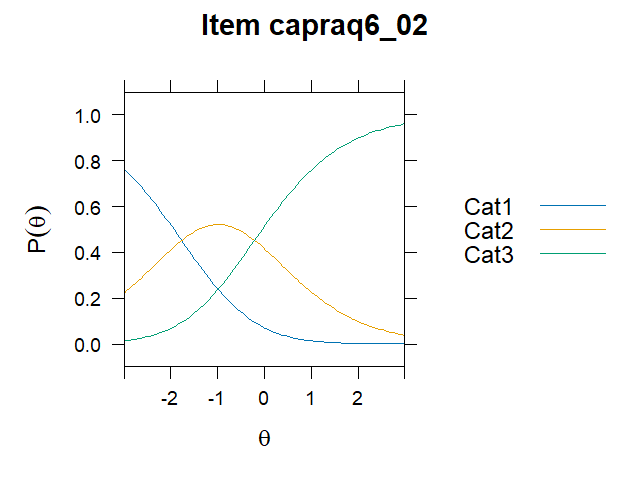 | 3f. | 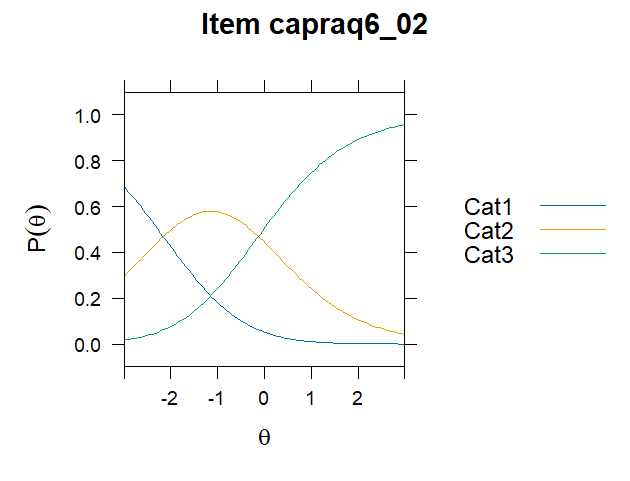 |
| 1g. | 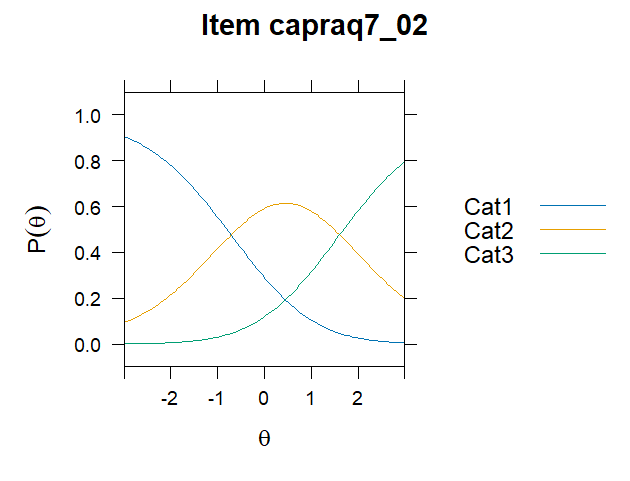 | 2g. | 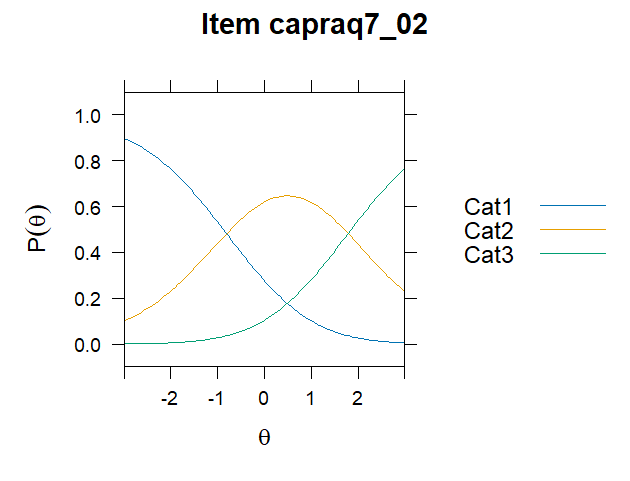 |  |  |
| 1h. | 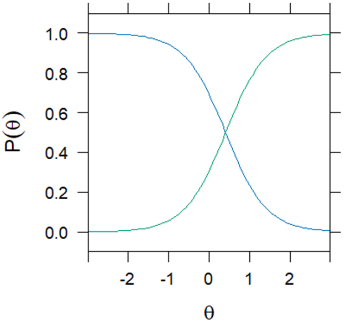 |  |  |  |  |
| **Supplementary Figure 1.1.** Category Characteristic Curves displaying the probability of answering each category (category 0 = blue, category 1 = yellow, category 2 = green), indicating correct ordering for CAP (1), CAP7 minus the pain distribution item (2), CAP6 minus the pain distribution and depression-associated items (3), for items associated with neuropathic-like pain (a); fatigue (b); cognitive impact (c); catastrophising (d); anxiety (e); sleep (f); depression (g); pain distribution (h). P(θ) probability to solve, θ = latent dimension. | | | | | |

|  | **Person-Item Map** | **Wright Map** |
| --- | --- | --- |
| **CAP** | 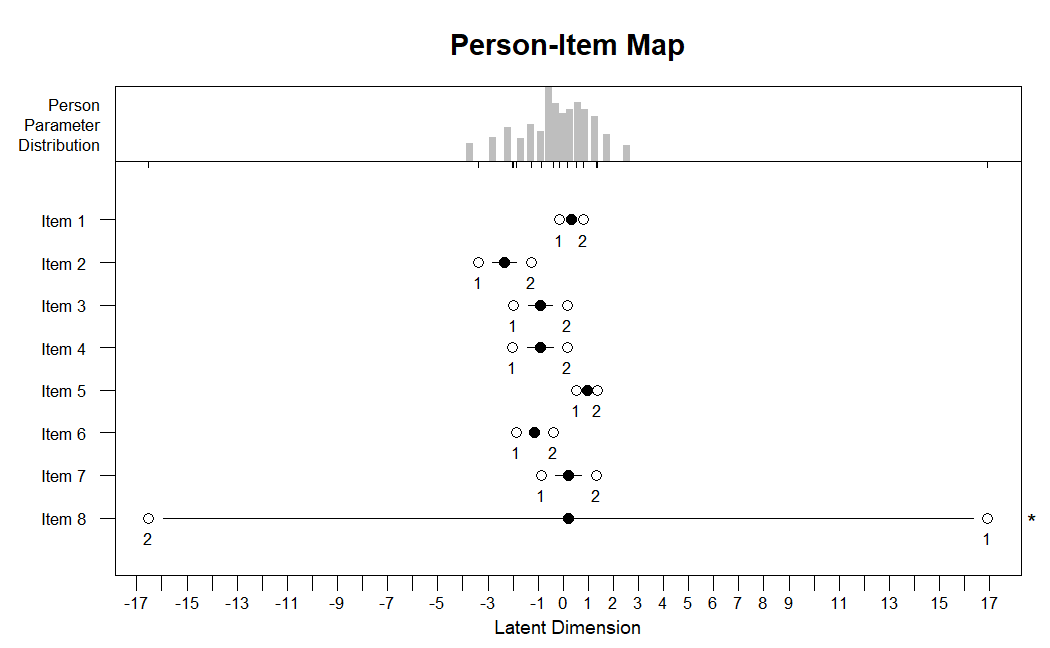 | 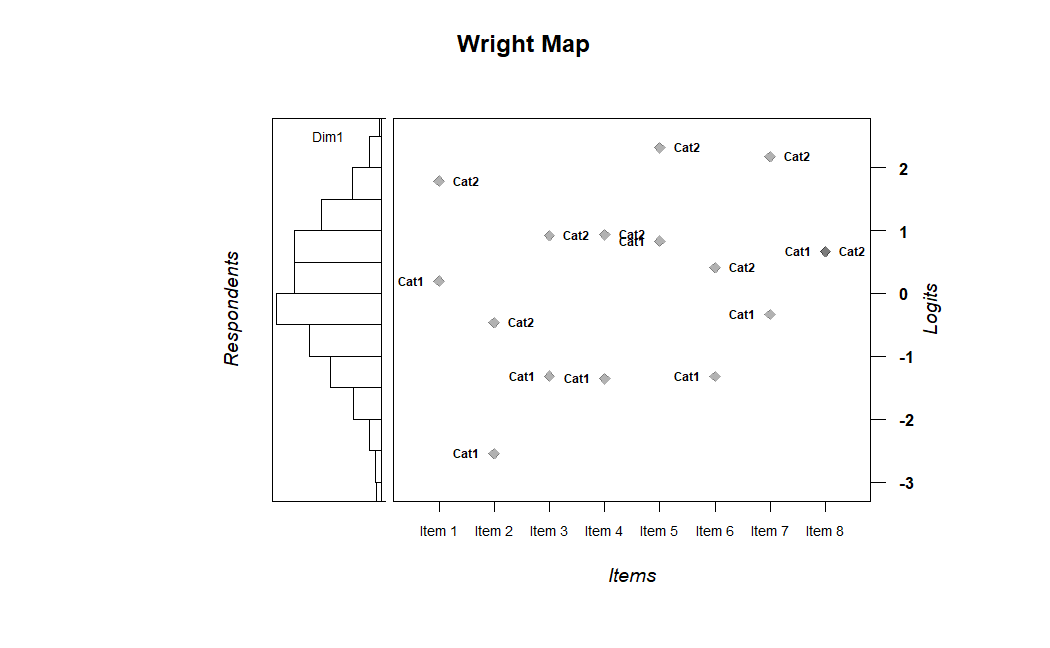 |
| **CAP7** | 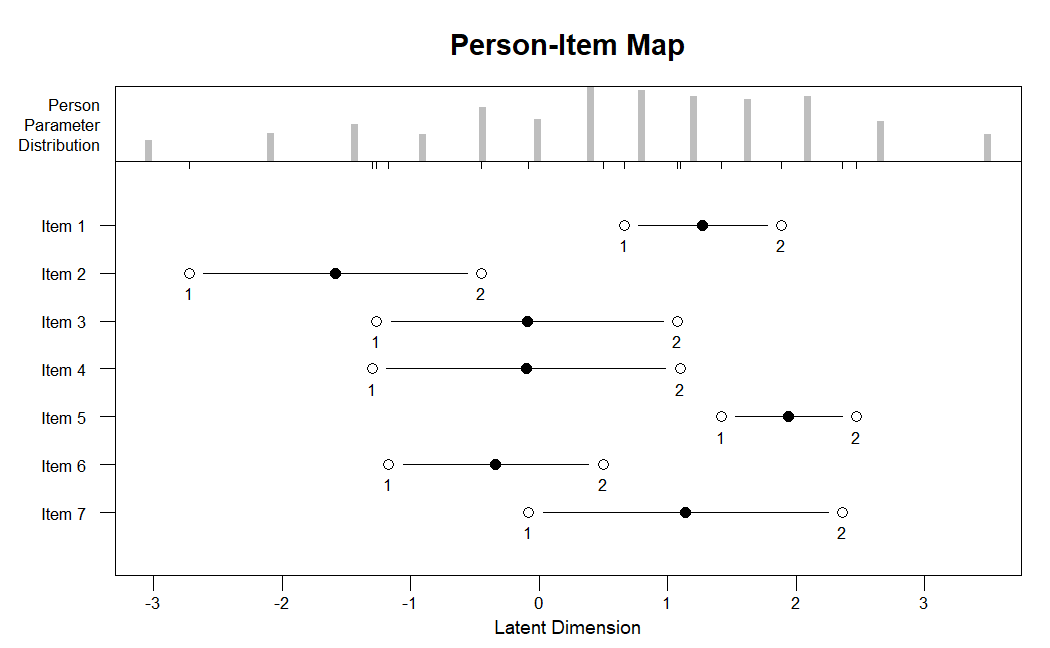 | 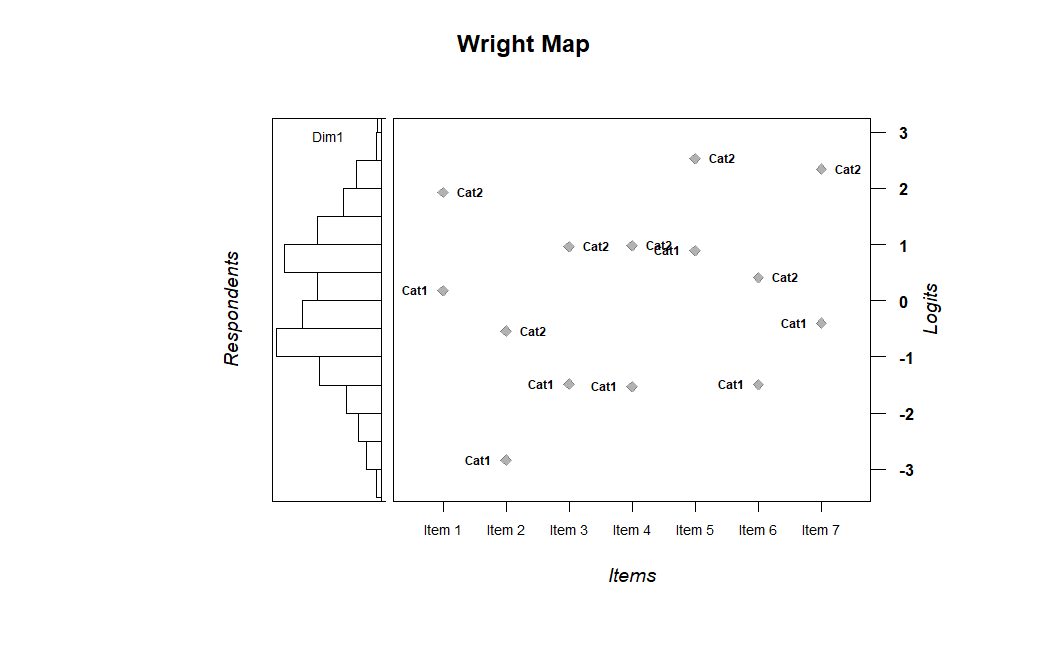 |
| **CAP6** | 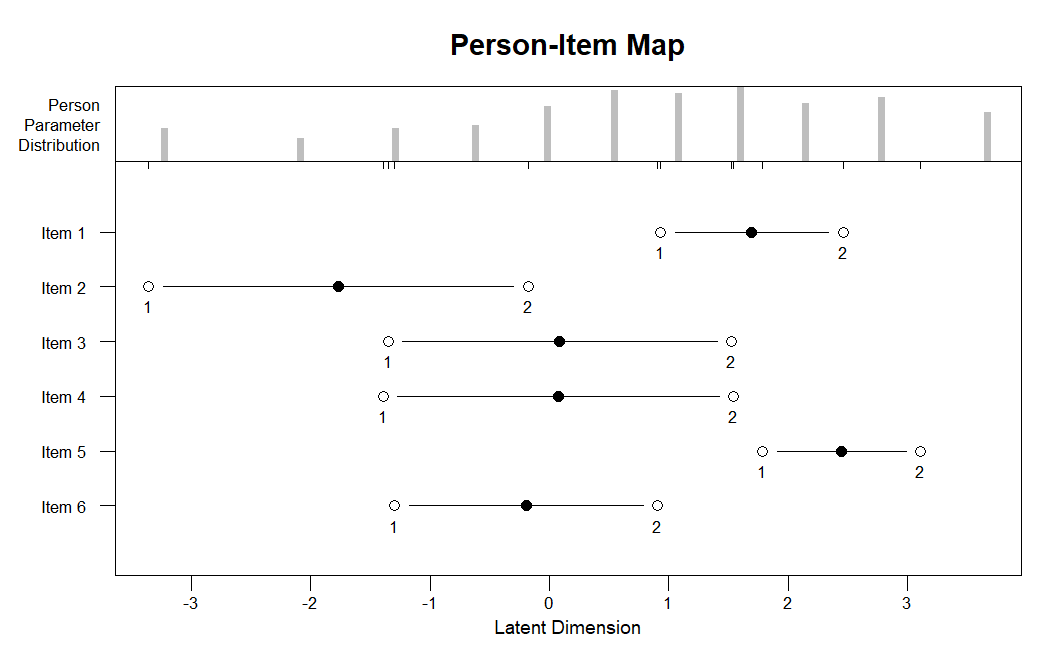 | 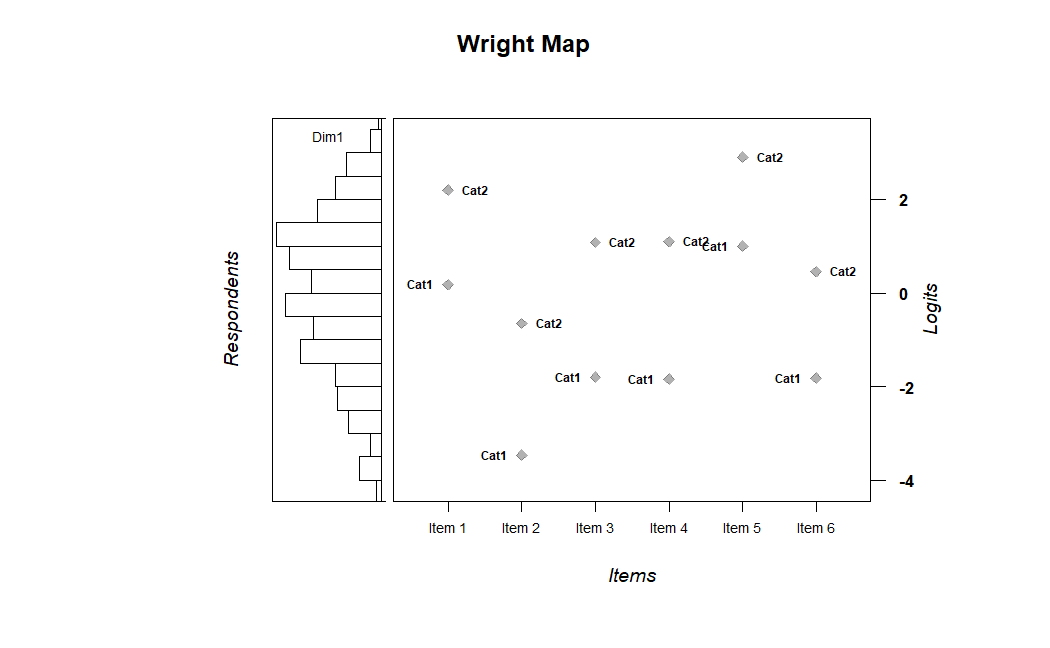 |
| **Supplementary Figure 1.2.** Person-item maps and Wright maps display the spread of item difficulties (solid circle) and item response threshold (hollow circles), demonstrating correct ordering for all items except in item 8 pain distribution in CAP. The ability of the person sample stretches over the entire range of item difficulties for CAP7 and CAP6, with most persons located at the majority of the items (no mistargeting). | | |

|  | **CAP** |  | **CAP7** |  | **CAP6** |  |
| --- | --- | --- | --- | --- | --- | --- |
| 1a. | 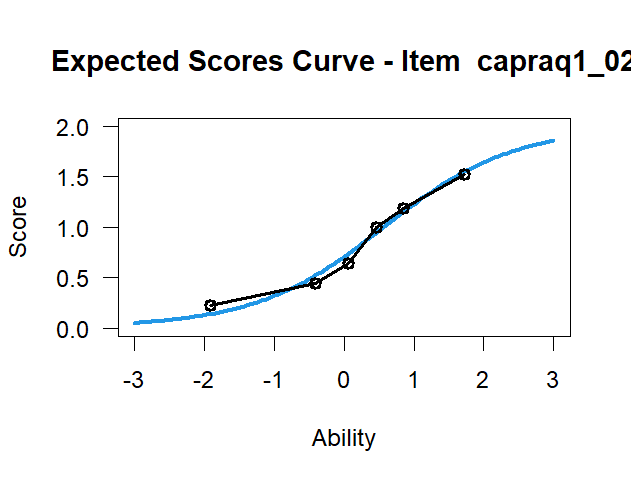 | 2a. | 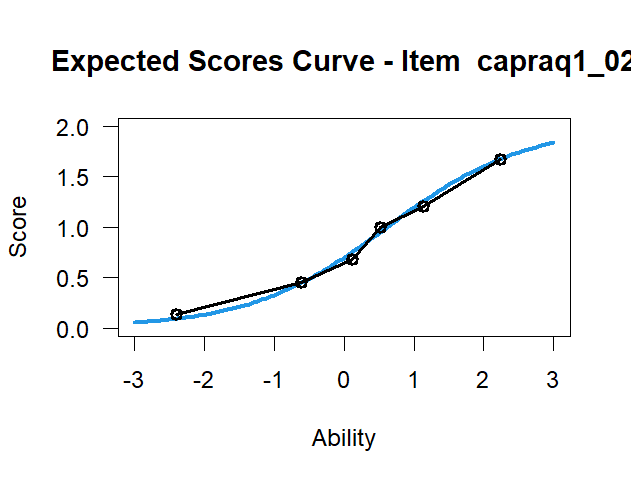 | 3a. | 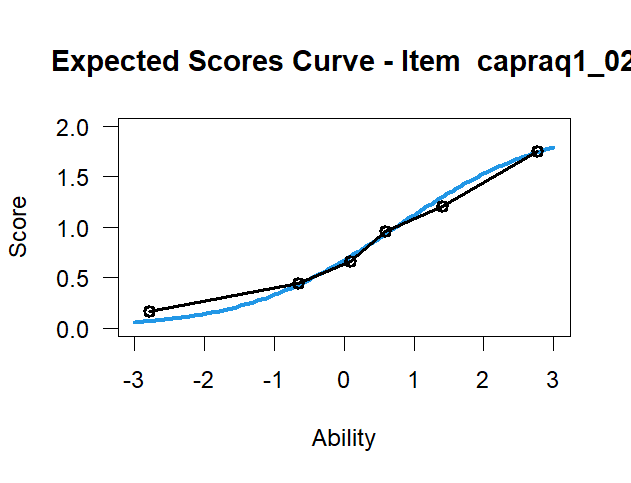 |  |
| 1b. | 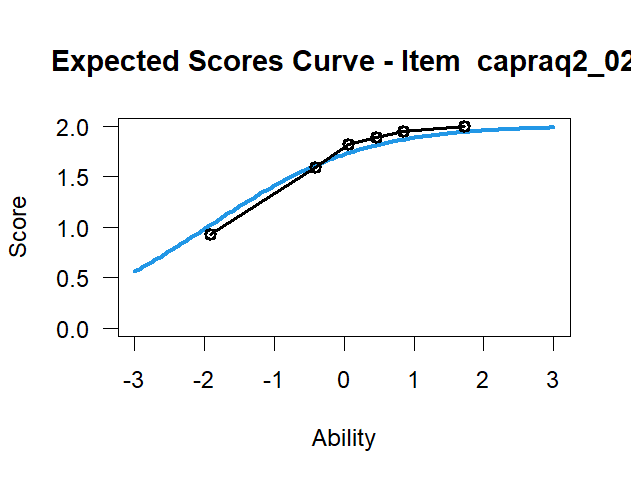 | 2b. | 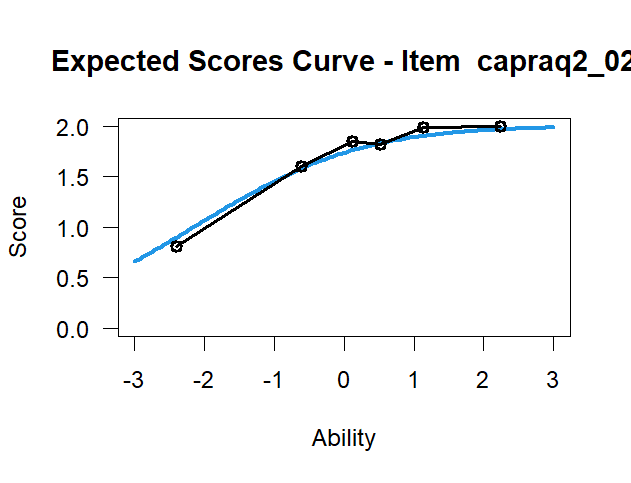 | 3b. | 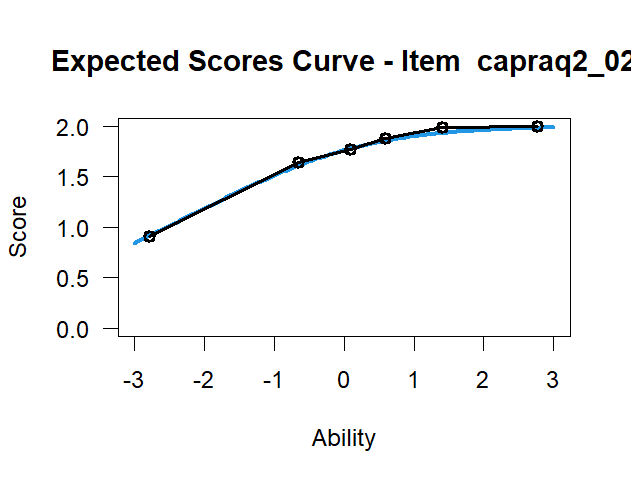 |  |
| 1c. | 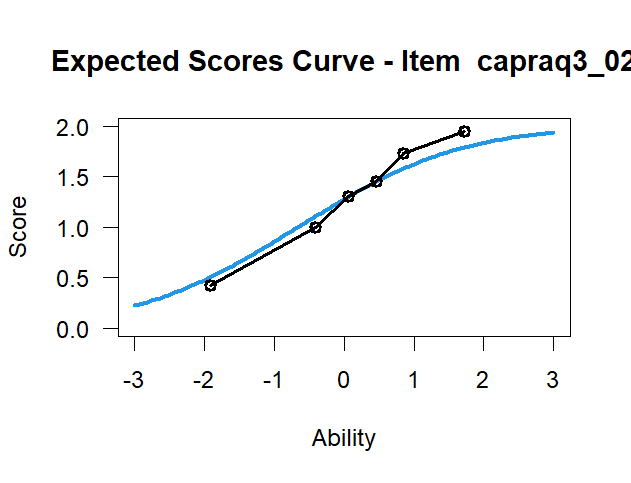 | 2c. | 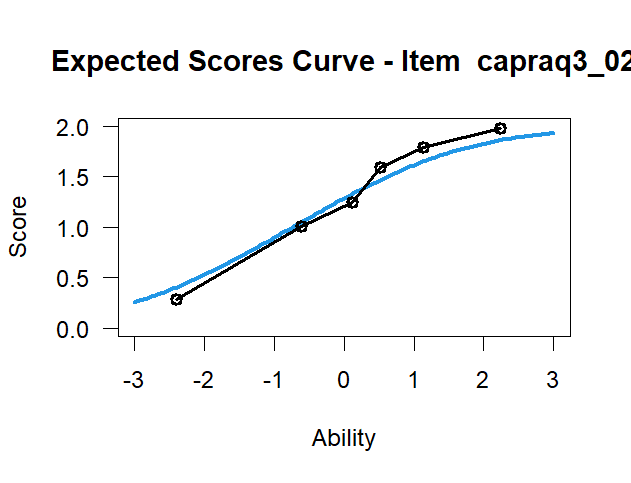 | 3c. | 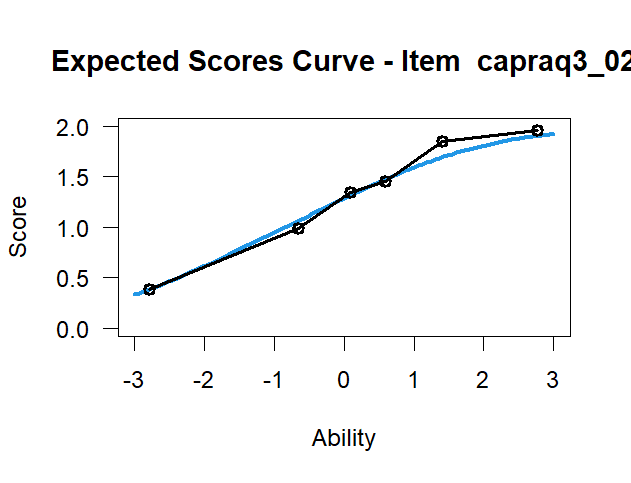 |  |
| 1d. | 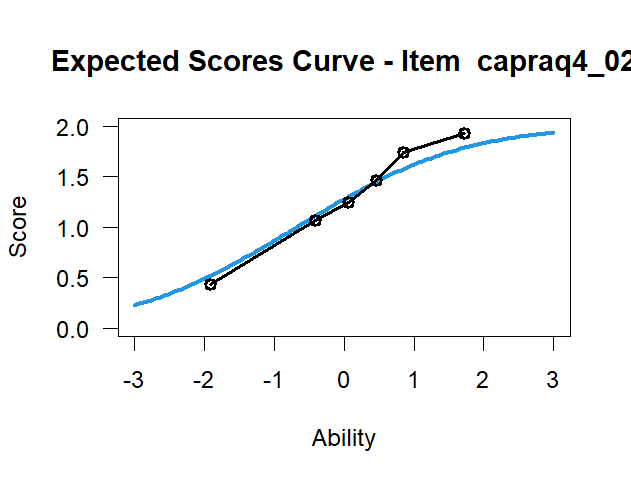 | 2d. | 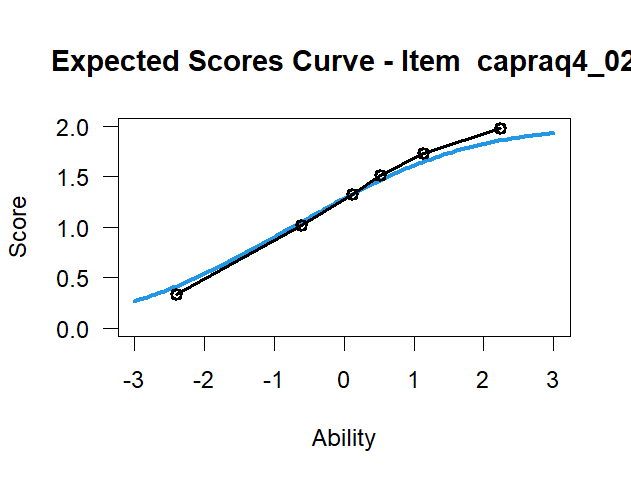 | 3d. | 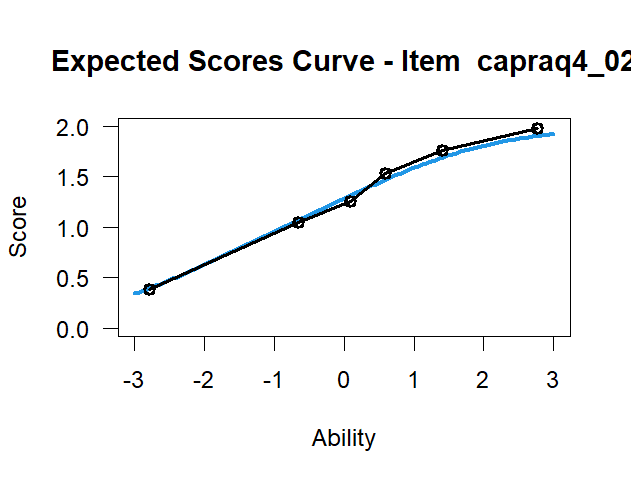 |  |
| 1e. | 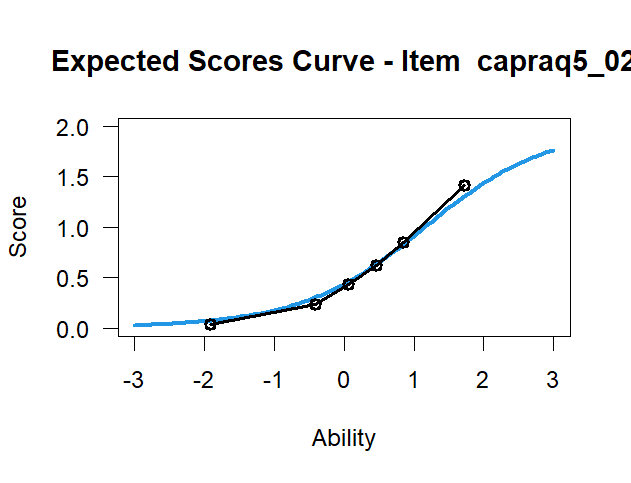 | 2e. | 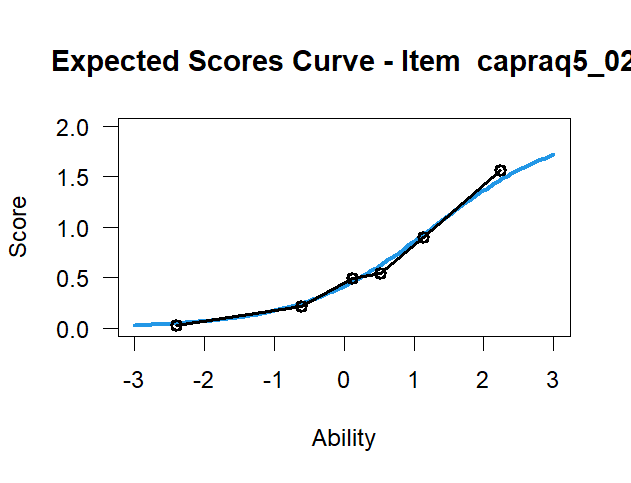 | 3e. | 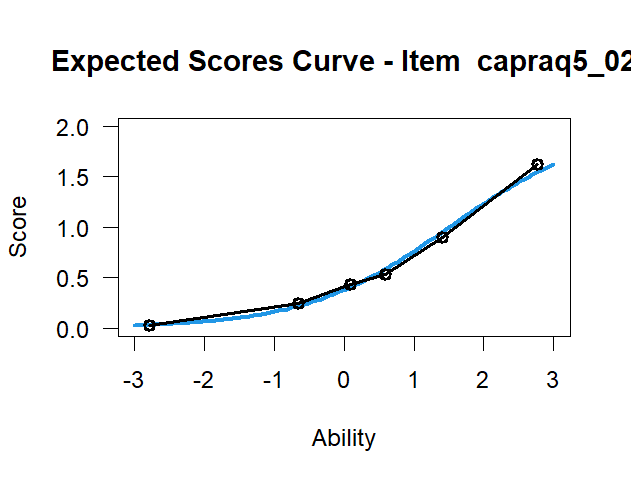 |  |
| 1f. | 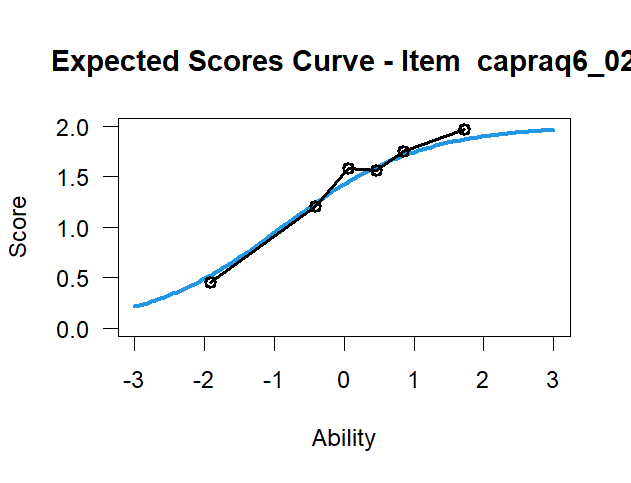 | 2f. | 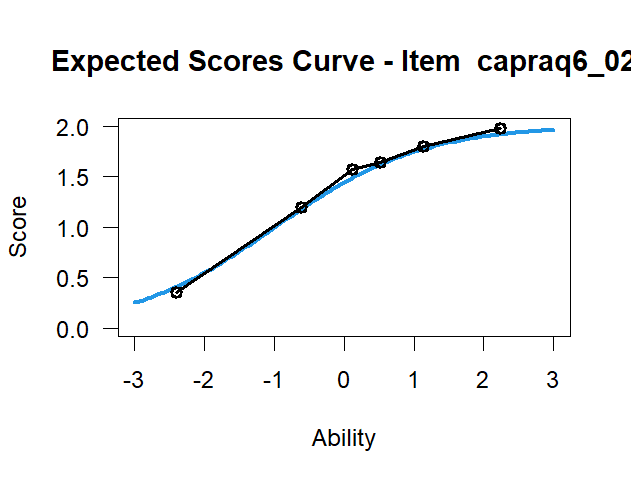 | 3f. | 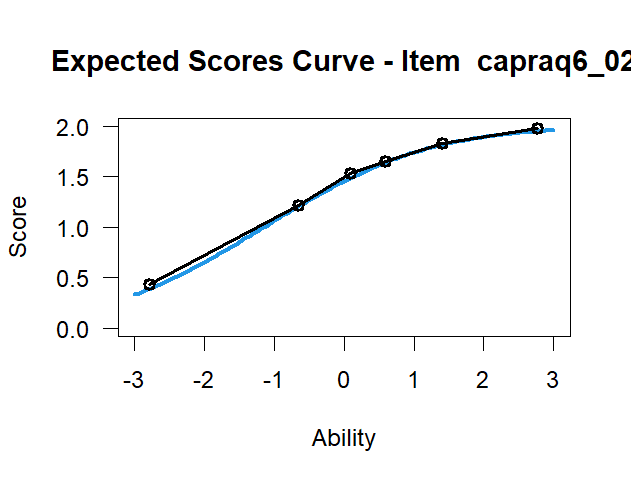 |  |
| 1g. | 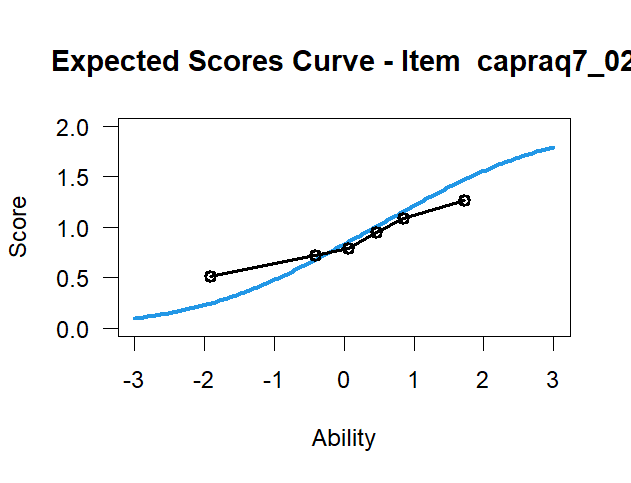 | 2g. | 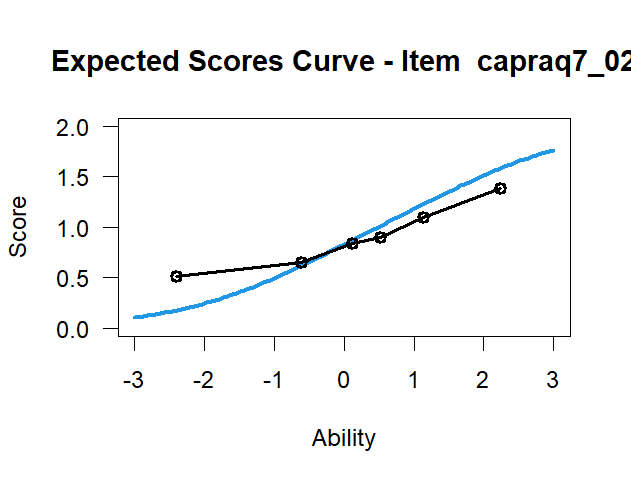 |  |  |  |
| 1h. | 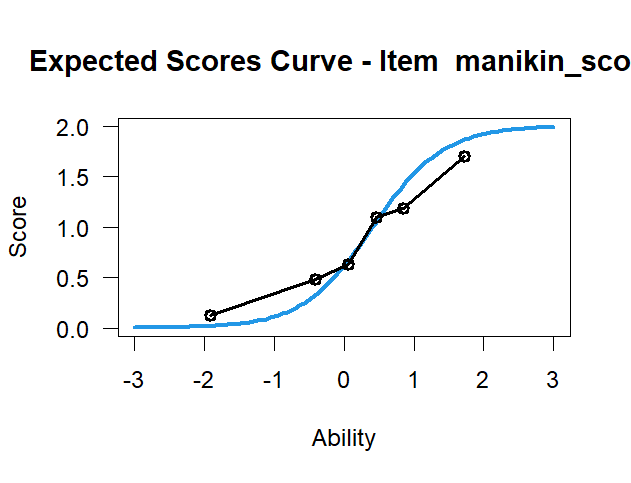 |  |  |  |  |  |
| **Supplementary Figure 1.3.** Empirical Item Characteristic Curves expected (blue solid line) observed (black line with points) for CAP (1), CAP7 minus the pain distribution item (2), CAP6 minus the pain distribution and depression-associated items (3), for items associated with neuropathic-like pain (a); fatigue (b); cognitive impact (c); catastrophising (d); anxiety (e); sleep (f); depression (g); pain distribution (h). | | | | | | |

| **Supplementary Table 1.4.** Fit statistics for CAP items | | | | | | | | | | | | |
| --- | --- | --- | --- | --- | --- | --- | --- | --- | --- | --- | --- | --- |
|  | **CAP** | | | | **CAP7** | | | | **CAP6** | | | |
| **Item label** | **Difficulty logit** | **SE logit** | **Outfit MNSQ** | **Infit MNSQ** | **Difficulty logit** | **SE logit** | **Outfit MNSQ** | **Infit MNSQ** | **Difficulty logit** | **SE logit** | **Outfit MNSQ** | **Infit MNSQ** |
| Neuropathic -like pain | 0.57 | 0.07 | 1.10 | 1.07 | 0.62 | 0.03 | 1.20 | 1.15 | 0.74 | 0.05 | 1.05 | 1.05 |
| Fatigue | -1.96 | 0.06 | 0.71 | 0.79 | -2.16 | 0.04 | 0.75 | 0.84 | -2.54 | 0.02 | 0.71 | 0.80 |
| Cognitive impact | -0.66 | 0.07 | 0.77 | 0.79 | -0.73 | 0.06 | 0.78 | 0.79 | -0.84 | 0.04 | 0.75 | 0.76 |
| Catastrophising | -0.67 | 0.06 | 0.80 | 0.81 | -0.75 | 0.04 | 0.80 | 0.83 | -0.86 | 0.03 | 0.76 | 0.78 |
| Anxiety | 1.16 | 0.02 | 0.95 | 0.99 | 1.28 | 0.01 | 1.03 | 1.04 | 1.51 | 0.02 | 0.93 | 0.98 |
| Sleep | -0.89 | 0.04 | 0.85 | 0.86 | -0.99 | 0.03 | 0.90 | 0.90 | -1.14 | 0.02 | 0.85 | 0.85 |
| Depression | 0.45 | 0.08 | **1.44** | **1.32** | 0.49 | 0.09 | **1.59** | **1.41** |  |  |  |  |
| Pain distribution | 0.41 | 0.09 | **2.03** | **1.37** |  |  |  |  |  |  |  |  |
| The fatigue-associated item was the easiest (lowest difficulty logit), and the anxiety-associated item was the most difficult (highest difficulty logit). Items associated with neuropathic like pain, fatigue, cognitive impact, catastrophising, anxiety and sleep demonstrate good fit (MNSQ within the range of 0.7 and 1.2). The depression-associated and pain distribution items demonstrate underfitting. N=380, **MNSQ:** Mean square residual, **SE:** standard error, **CAP:** Central Aspects of Pain, **CAP7:** Central Aspects of Pain minus pain distribution item; **CAP6:** Central Aspects of Pain minus pain distribution and depression items. Item labels indicate psychological or symptom constructs that have been associated with the individual items included within the CAP questionnaire. The single items within CAP should not be taken to represent reliable measures of those constructs. | | | | | | | | | | | | |

| **Supplementary Table 1.5.** Differential Item Functioning (DIF) between sexes (Male, Female) | | | | | | |
| --- | --- | --- | --- | --- | --- | --- |
|  | Uniformed DIF | | Non-Uniformed DIF | | Change between Uniformed and Non-Uniformed DIF | |
| Item label | Χ^2^ p-value | Pseudo R^2^ | Χ^2^ p-value | Pseudo R^2^ | Χ^2^ p-value | Pseudo R^2^ |
| Neuropathic -like pain | 0.002 | -0.004 | 0.07 | -0.003 | 0.594 | 0.001 |
| Fatigue | 0.020 | -0.009 | 0.049 | -0.008 | 0.437 | 0.001 |
| Cognitive impact | 0.010 | -0.011 | 0.032 | -0.011 | 0.604 | <0.001 |
| Catastrophising | 0.022 | -0.010 | 0.008 | -0.004 | 0.038 | 0.006 |
| Anxiety | 0.021 | -0.014 | 0.020 | -0.009 | 0.111 | 0.005 |
| Sleep | 0.052 | -0.018 | 0.066 | -0.015 | 0.198 | 0.003 |
| Depression | 0.002 | -0.015 | 0.008 | -0.014 | 0.479 | 0.001 |
| Pain distribution | 0.028 | -0.006 | 0.049 | -0.003 | 0.266 | 0.003 |
| No differences were observed between sexes (χ^2^ nonsignificant or pseudo R^2^ <0.035). Item labels indicate psychological or symptom constructs that have been associated with the individual items included within the CAP questionnaire. The single items within CAP should not be taken to represent reliable measures of those constructs. | | | | | | |

| **Supplementary Table 1.6.** Differential Item Functioning (DIF) between ages (<65, ≥65 years) | | | | | | |
| --- | --- | --- | --- | --- | --- | --- |
|  | Uniformed DIF | | Non-Uniformed DIF | | Change between Uniformed and Non-Uniformed DIF | |
| Item label | Χ^2^ p-value | Pseudo R^2^ | Χ^2^ p-value | Pseudo R^2^ | Χ^2^ p-value | Pseudo R^2^ |
| Neuropathic -like pain | <0.001 | -0.003 | <0.001 | -0.003 | 0.841 | <0.001 |
| Fatigue | 0.056 | -0.009 | 0.104 | -0.008 | 0.352 | 0.002 |
| Cognitive impact | <0.001 | -0.002 | <0.001 | -0.001 | 0.394 | 0.001 |
| Catastrophising | <0.001 | -0.006 | 0.001 | -0.006 | 0.650 | <0.001 |
| Anxiety | 0.001 | -0.020 | 0.005 | -0.019 | 0.805 | <0.001 |
| Sleep | <0.001 | 0.005 | <0.001 | 0.008 | 0.185 | 0.003 |
| Depression | <0.001 | -0.011 | <0.001 | -0.011 | 0.689 | <0.001 |
| Pain distribution | <0.001 | 0.004 | 0.001 | 0.006 | 0.412 | 0.003 |
| No differences were observed between ages (χ^2^ nonsignificant or pseudo R^2^ <0.035). Item labels indicate psychological or symptom constructs that have been associated with the individual items included within the CAP questionnaire. The single items within CAP should not be taken to represent reliable measures of those constructs. | | | | | | |

| **Supplementary Table 1.7.** Differential Item Functioning (DIF) between sites (Nottingham, Cardiff, London) | | | | | | |
| --- | --- | --- | --- | --- | --- | --- |
|  | Uniformed DIF | | Non-Uniformed DIF | | Change between Uniformed and Non-Uniformed DIF | |
| Item label | Χ^2^ p-value | Pseudo R^2^ | Χ^2^ p-value | Pseudo R^2^ | Χ^2^ p-value | Pseudo R^2^ |
| Neuropathic -like pain | 0.538 | 0.003 | 0.286 | 0.010 | 0.151 | 0.008 |
| Fatigue | 0.189 | 0.006 | 0.356 | 0.008 | 0.591 | 0.002 |
| Cognitive impact | 0.173 | 0.005 | 0.103 | 0.010 | 0.124 | 0.006 |
| Catastrophising | 0.316 | 0.003 | 0.550 | 0.004 | 0.689 | 0.001 |
| Anxiety | 0.187 | 0.007 | 0.347 | 0.009 | 0.572 | 0.002 |
| Sleep | 0.213 | 0.005 | 0.065 | 0.014 | 0.057 | 0.009 |
| Depression | 0.344 | 0.006 | <0.001 | 0.101 | <0.001 | 0.095 |
| Pain distribution | 0.005 | 0.028 | 0.007 | 0.036 | 0.200 | 0.008 |
| Large non-uniformed DIF and change between uniformed and non-uniformed DIF were displayed for the depression-associated item (χ^2^ significant and pseudo R^2^ >0.070). A moderate non-uniformed DIF was displayed for pain distribution (χ^2^ significant and pseudo R^2^ (0.035 to 0.007). There were no differences observed between sites for the remaining 6 items (χ^2^ insignificant or pseudo R^2^ <0.035). Item labels indicate psychological or symptom constructs that have been associated with the individual items included within the CAP questionnaire. The single items within CAP should not be taken to represent reliable measures of those constructs. | | | | | | |

## Reliability

A total of 24 people returned a second (repeat) CAP questionnaire with a median of 10 (IQR: 8.3 to 14; range 7 to 27) days after their initial questionnaire. Reliability was deemed acceptable (ICC_(3,1)_ 0.86 (95% CI 0.75 to 0.94). Removing the pain distribution item didn’t affect the reliability (ICC_(3,1)_ 0.89 (95% CI 0.77 to 0.94).

## Associations of CAP and CAP7 with pain and linked traits

CAP scores demonstrated significant moderate associations with pain, weakly associated with SJC and non-significant associations with QST. Associations with pain remained significant when adjusted for age, sex, and BMI (Supplementary Table 1.8). Removing the pain distribution item (CAP7) did not affect the associations.

Individual CAP item scores were associated with questionnaires addressing fatigue, cognitive impact, catastrophising, anxiety, sleep, depression and widespread pain (Supplementary Table 1.9). Removing the pain distribution item (CAP7) did not affect the associations.

Summated pain score was associated with CRP and SJC, and these inflammation indices together explained 10% of pain variance (Supplementary Table 1.10). The inclusion of the CAP score explained an additional 28% of pain variance, and a full model, including age, sex, BMI, SJC, CRP and CAP-RA score, explained 42% of pain variance (Supplementary Table 1.10). Removing the pain distribution item (CAP7) did not affect the associations.

| **Supplementary Table 1.8.** Individual bivariate (unadjusted model) and multivariable (adjusted model, for age, sex and BMI) linear regression models of associations between Central Aspects of Pain (CAP) or CAP minus pain distribution item (CAP7) scores and pain, pain sensitivity or inflammation. | | | | | | |
| --- | --- | --- | --- | --- | --- | --- |
|  | **CAP unadjusted model** | | | **CAP7 unadjusted model** | | |
|  | **Β** | **95% CI** | **p-value** | **β** | **95% CI** | **p-value** |
| **Pain (n=343)** | | | | | | |
| Combined pain | 0.57 | 0.48, 0.66 | <0.001 | 0.57 | 0.49, 0.66 | <0.001 |
| Pain now | 0.50 | 0.41, 0.59 | <0.001 | 0.50 | 0.40, 0.59 | <0.001 |
| Strongest pain past 4 weeks | 0.55 | 0.46, 0.64 | <0.001 | 0.55 | 0.46, 0.64 | <0.001 |
| Average pain past 4 weeks | 0.53 | 0.43, 0.63 | <0.001 | 0.53 | 0.43, 0.63 | <0.001 |
| Modified painDETECT | 0.56 | 0.47, 0.66 | <0.001 | 0.58 | 0.48, 0.67 | <0.001 |
| **Pain sensitivity (n=90)** | | | | | | |
| PPT medial joint line | -0.05 | -0.25, 0.15 | 0.606 | -0.03 | -0.22, 0.16 | 0.760 |
| PPT Brachioradialis | -0.001 | -0.19, 0.18 | 0.985 | 0.04 | -0.14, 0.21 | 0.694 |
| PPT Tibialis anterior | -0.07 | -0.25, 0.12 | 0.460 | -0.03 | -0.21, 0.15 | 0.737 |
| TSP | 0.06 | -0.13, 0.25 | 0.519 | 0.05 | -0.13, 0.23 | 0.570 |
| CPM | -0.04 | -0.22, 0.15 | 0.692 | 0.00 | -0.18, 0.18 | 0.967 |
| **Inflammation (n=90)** | | | | | | |
| SJC | 0.21 | 0.28, 0.39 | 0.024 | 0.18 | -0.01, 0.36 | 0.055 |
| CRP | 0.12 | -0.06, 0.31 | 0.194 | 0.11 | -0.09, 0.30 | 0.279 |
|  | **CAP adjusted model** | | | **CAP7 adjusted model** | | |
|  | **Β** | **95% CI** | **p-value** | **β** | **95% CI** | **p-value** |
| **Pain (n=343)** | | | | | | |
| Combined pain | 0.75 | 0.54, 0.96 | <0.001 | 070 | 0.50, 0.90 | <0.001 |
| Pain now | 0.52 | 0.31, 0.73 | <0.001 | 0.53 | 0.34, 0.73 | <0.001 |
| Strongest pain past 4 weeks | 0.69 | 0.47, 0.91 | <0.001 | 0.61 | 0.40, 0.83 | <0.001 |
| Average pain past 4 weeks | 0.65 | 0.46, 0.83 | <0.001 | 0.58 | 0.40, 0.76 | <0.001 |
| Modified painDETECT | 0.58 | 0.40, 0.76 | <0.001 | 0.54 | 0.37, 0.71 | <0.001 |
| **Pain sensitivity (n=90)** | | | | | | |
| PPT medial joint line | -0.06 | -0.26, 0.15 | 0.602 | -0.03 | -0.23, 0.16 | 0.747 |
| PPT Brachioradialis | -0.01 | -0.21, 0.19 | 0.915 | 0.03 | -0.16, 0.21 | 0.776 |
| PPT Tibialis anterior | -0.07 | -0.28, 0.13 | 0.470 | -0.03 | -0.22, 0.16 | 0.767 |
| TSP | 0.07 | -0.12, 0.27 | 0.459 | 0.07 | -0.12, 0.26 | 0.470 |
| CPM | -0.06 | -0.25, 0.14 | 0.570 | -0.02 | -0.21, 0.16 | 0.802 |
| **Inflammation (n=90)** | | | | | | |
| SJC | 0.23 | 0.04, 0.42 | 0.016 | 0.18 | 0.01, 0.36 | 0.045 |
| CRP | 0.19 | -0.01, 0.38 | 0.064 | 0.13 | -0.05, 0.32 | 0.162 |
| **Β:** Standardised beta coefficient; **CAP:** Central Aspects of Pain; **CAP7:** Central Aspects of Pain minus pain distribution item; **CPM:** Conditioned Pain Modulation; **CRP:** C-reactive protein; **PPT:** Pressure Pain detection Threshold; **SJC:** Swollen Joint Count; **TSP:** Temporal Summation of Pain; ; **modified painDETECT**: pain detect score with item 6 removed due to inclusion in CAP; **95% CI:** 95% confidence interval. | | | | | | |

| **Supplementary Table 1.9.** Associations between Central Aspects of Pain (CAP) and related characteristics | | | | | |
| --- | --- | --- | --- | --- | --- |
| **Characteristic** | **Questionnaire** | **Unadjusted** | | **Adjusted** | |
|  |  | **Β (95% CI)** | **P value** | **Β (95% CI)** | **P value** |
| **CAP** |  |  |  |  |  |
| Neuropathic -like pain | painDETECT | 0.55 (0.45, 0.65) | <0.001 | 0.49 (0.30, 0.69) | <0.001 |
| Fatigue | BRAF total fatigue | 0.73 (0.64, 0.82) | <0.001 | 0.70 (0.56, 0.83) | <0.001 |
| Cognitive impact | CFQ total | -0.36 (-0.52, -0.19) | <0.001 | -0.38 (-0.57, -0.19) | 0.001 |
| Catastrophising | PCS total | 0.57 (0.42, 0.71) | <0.001 | 0.58 (0.41, 0.74) | <0.001 |
| Anxiety | HADs Anxiety | 0.43 (0.28, 0.58) | <0.001 | 0.43 (0.27, 0.59) | <0.001 |
| Sleep | AIS | 0.50 (0.34, 0.65) | <0.001 | 0.51 (0.35, 0.68) | <0.001 |
| Depression | HADS depression | 0.45 (0.31, 0.59) | <0.001 | 0.48 (0.32, 0.64) | <0.001 |
| Pain distribution | WPI | 0.26 (0.09, 0.43) | 0.002 | 0.29 (0.11, 0.46) | 0.001 |
| Pain distribution | Fibromyalgianess score | 0.38 (0.22, 0.23) | <0.001 | 0.42 (0.26, 0.58) | <0.001 |
| **CAP7** |  |  |  |  |  |
| Neuropathic -like pain | painDETECT | 0.55 (0.45, 0.64) | <0.001 | 0.49 (0.31, 0.68) | <0.001 |
| Fatigue | BRAF total fatigue | 0.70 (0.61, 0.79) | <0.001 | 0.66 (0.53, 0.78) | <0.001 |
| Cognitive impact | CFQ total | -0.37 (-0.54, -0.21) | <0.001 | -0.38 (-0.56, -0.21) | <0.001 |
| Catastrophising | PCS total | 0.56 (0.42, 0.70) | <0.001 | 0.56 (0.41, 0.71) | <0.001 |
| Anxiety | HADs Anxiety | 0.40 (0.25, 0.56) | <0.001 | 0.40 (0.24, 0.56) | <0.001 |
| Sleep | AIS | 0.51 (0.34, 0.66) | <0.001 | 0.52 (0.38, 0.68) | <0.001 |
| Depression | HADS depression | 0.50 (0.36, 0.64) | <0.001 | 0.53 (0.38, 0.69) | <0.001 |
| Standardised beta coefficients are derived from separate multivariable linear regression models with the CAP. CAP scores were modified by omission of the item that was originally derived from a questionnaire addressing that characteristic tested by the model. For example, the CAP fatigue item was omitted from CAP scores in linear regression against BRAF fatigue scores. Models are presented unadjusted and adjusted for age, sex and BMI.  **AIS:** Athens Insomnia Scale; **B:** Standardised Beta; **BRAF:** Bristol Rheumatoid Arthritis Fatigue Scale; ; **CAP:** Central Aspects of Pain; **CAP7:** Central Aspects of Pain minus pain distribution item; **CFQ:** Cognitive failures questionnaire; **HADs:** Hospital Anxiety and Depression scale; **painDETECT:** total score from modified painDETECT questionnaire; **PCS:** pain catastrophising scale; **SE:** Standard Error; **WPI:** Widespread pain index (manikin score). | | | | | |

| **Supplementary Table 1.10.** Multivariable models for contributions of inflammation and Central Aspects of Pain to summated pain scores | | | | | | |
| --- | --- | --- | --- | --- | --- | --- |
|  | **B** | **95% CI** | **p-value** |  |  |  |
| **Model 1 (inflammation)** | | | | | | |
| CRP | 0.23 | 0.01, 0.43 | 0.038 |  |  |  |
| SJC | 0.24 | 0.03, 0.45 | 0.027 |  |  |  |
|  | R^2^=0.097, p=0.017 | | |  | | |
|  | **CAP** | | | **CAP7** | | |
|  | **B** | **95% CI** | **p-value** | **B** | **95% CI** | **p-value** |
| **Model 2 (inflammation and CAP or CSI-9)** | | | | | | |
| CRP | 0.10 | -0.08, 0.28 | 0.283 | 0.13 | -0.05, 0.32 | 0.142 |
| SJC | 0.08 | -0.10, 0.27 | 0.376 | 0.11 | -0.07, 0.30 | 0.217 |
| CAP/CAP7 | 0.56 | 0.38, 0.75 | <0.001 | 0.55 | 0.36, 0.73 | <0.001 |
|  | R^2^=0.378, p<0.001 | | | R^2^=0.376, p<0.001 | | |
| **Model 3 (inflammation and CAP or CSI-9, fully adjusted)** | | | | | | |
| CRP | 0.09 | -0.09, 0.28 | 0.301 | 0.13 | -0.05, 0.31 | 0.154 |
| SJC | 0.11 | -0.07, 0.30 | 0.222 | 0.15 | -0.04, 0.33 | 0.114 |
| CAP/CAP7 | 0.58 | 0.39, 0.77 | <0.001 | 0.57 | 0.38, 0.75 | <0.001 |
| Age | 0.12 | -0.06, 0.30 | 0.175 | 0.14 | -0.04, 0.32 | 0.125 |
| Sex | 0.13 | -0.05, 0.31 | 0.145 | 0.12 | -0.06, 0.30 | 0.186 |
| BMI | -0.09 | -0.27, 0.08 | 0.298 | 0.10 | -0.28, 0.08 | 0.257 |
|  | R^2^=0.419, p<0.001 | | | R^2^=0.419, p<0.001 | | |
| N=83. Step-wise multivariable linear regression models explaining summated pain scores. **B:** Standardised Beta; **BMI:** Body Mass Index; **CAP:** Central Aspects of Pain; **CRP:** C-Reactive Protein; **CAP7:** Central Aspects of Pain minus pain distribution item; **SJC:** Swollen Joint Count; **95% CI:** 95% Confidence Interval | | | | | | |
